# Biohybrid Nanorobots Carrying Glycoengineered Extracellular Vesicles Promote Diabetic Wound Repair through Dual‐Enhanced Cell and Tissue Penetration

**DOI:** 10.1002/advs.202404456

**Published:** 2024-06-18

**Authors:** Chengqi Yan, Kai Feng, Bingkun Bao, Jing Chen, Xiang Xu, Guoyong Jiang, Yufeng Wang, Jiahe Guo, Tao Jiang, Yu Kang, Cheng Wang, Chengcheng Li, Chi Zhang, Pengjuan Nie, Shuoyuan Liu, Hans‐Günther Machens, Linyong Zhu, Xiaofan Yang, Ran Niu, Zhenbing Chen

**Affiliations:** ^1^ Department of Hand Surgery Union Hospital Tongji Medical College Huazhong University of Science and Technology Wuhan 430022 China; ^2^ Key Laboratory of Material Chemistry for Energy Conversion and Storage Ministry of Education School of Chemistry and Chemical Engineering Huazhong University of Science and Technology Wuhan 430074 China; ^3^ School of Biomedical Engineering Shanghai Jiao Tong University Shanghai 200240 China; ^4^ Department of Dermatology Wuhan No.1 Hospital Wuhan Hubei 430022 China; ^5^ Department of Plastic and Hand Surgery Technical University of Munich D‐80333 Munich Germany

**Keywords:** biohybrid nanorobots, diabetic wound, drug delivery, extracellular vesicles, glycoengineering

## Abstract

Considerable progress has been made in the development of drug delivery systems for diabetic wounds. However, underlying drawbacks, such as low delivery efficiency and poor tissue permeability, have rarely been addressed. In this study, a multifunctional biohybrid nanorobot platform comprising an artificial unit and several biological components is constructed. The artificial unit is a magnetically driven nanorobot surface modified with antibacterial 2‐hydroxypropyltrimethyl ammonium chloride chitosan, which enables the entire platform to move and has excellent tissue penetration capacity. The biological components are two‐step engineered extracellular vesicles that are first loaded with mangiferin, a natural polyphenolic compound with antioxidant properties, and then glycoengineered on the surface to enhance cellular uptake efficiency. As expected, the platform is more easily absorbed by endothelial cells and fibroblasts and exhibits outstanding dermal penetration performance and antioxidant properties. Encouraging results are also observed in infected diabetic wound models, showing improved wound re‐epithelialization, collagen deposition, angiogenesis, and accelerated wound healing. Collectively, a biohybrid nanorobot platform that possesses the functionalities of both artificial units and biological components serves as an efficient delivery system to promote diabetic wound repair through dual‐enhanced cell and tissue penetration and multistep interventions.

## Graphical abstract

A biohybrid nanorobot platform combining a magnetically driven nanorobot surface modified with 2‐hydroxypropyltrimethyl ammonium chloride chitosan and glycoengineered extracellular vesicles loaded with mangiferin is developed. It alleviates oxidative stress, improves endothelial cell and fibroblast functions, and promotes wound re‐epithelialization, collagen deposition, and angiogenesis through dual‐enhanced cell and tissue penetration, thus showing great promise for diabetic wound repair.

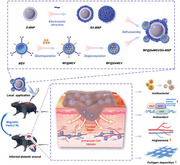

## 1 Introduction

As a chronic diabetic complication, non‐healing wounds cause severe physiological and psychological pain in patients and are a huge burden to society. Diabetic wounds are multifactorial, and oxidative stress damage is one of the most widely accepted mechanisms in the development of the disease.^[^ ^1^, ^2^ ^]^ Abnormally increased reactive oxygen species (ROS) cannot be scavenged by the endogenous antioxidant system, thus causing oxidative damage to the biomacromolecules in cells and ultimately leading to tissue dysfunction. In addition, diabetic wounds are more susceptible to bacterial colonization, which hinders the process of wound repair and eventually evolves into chronically infected wounds.^[^ ^3^ ^]^ Though a variety of wound‐healing dressings have been developed to treat diabetic wounds, most of these approaches are based on the passive diffusion of therapeutic agents, resulting in low delivery efficiency and weak drug permeability at the wound site.^[^ ^4^, ^5^ ^]^ Therefore, there is an urgent need to design a wound healing platform that can facilitate the infiltration of drugs to the deep and surrounding wound tissue.

Recently, micro/nanorobots (MNRs) capable of autonomous movement have shown great potential for development in various biomedical fields, including drug delivery, minimally invasive surgery, imaging‐guided therapy, and biofilm disruption.^[^ ^6^, ^7^ ^]^ These micro or nanoscale motors can convert chemical or external energy (such as magnetic, electrical, acoustic, and optical) into their own kinetic energy on demand for various tasks. Compared to traditional passive cargo delivery carriers, which often lack active movement ability, MNRs can overcome multiple physiological barriers, thus significantly enhancing bioadhesion, tissue penetration, and cargo retention.^[^ ^8^ ^]^ Further, biological components such as cells, bacteria, algae, and enzymes can be combined with synthetic MNRs to improve their performance. These biohybrid MNRs, which possess the functionalities of both biological elements and synthetic units, provide a promising strategy for achieving advanced capabilities with greater precision and accuracy.^[^ ^9^, ^10^ ^]^ In areas where enhanced penetration is required, such as wound repair, MNRs should exhibit more effective therapy, but very few studies were reported using MNRs.

Extracellular vesicles (EVs) are natural nano‐sized particles with lipid‐bilayer enclosures that are secreted from cells to regulate various physiological and pathological processes. In addition to their biological functions, EVs exhibit great potential as next‐generation drug delivery systems owing to their unique advantages, such as low immunogenicity, good biocompatibility, and strong protective effects on therapeutic contents.^[^ ^11^ ^]^ EV surface glycans are crucial biomolecules present on EV membranes and are complex and diverse, including N‐glycans, O‐glycans, heparan sulfate proteoglycans (HSPGs), and so on.^[^ ^12^, ^13^ ^]^ Many studies investigating their effects on EV biogenesis and cell‐EV interaction, as well as their applications as EV subpopulation indicators and disease biomarkers have emerged.^[^ ^14^, ^15^, ^16^, ^17^ ^]^ Based on these early studies, researchers have recognized that engineering of EV surface glycans can be a useful strategy for regulating the cellular recognition and uptake of EVs to improve EV‐mediated drug delivery efficiency.^[^ ^15^, ^18^ ^]^

**Figure 1:**
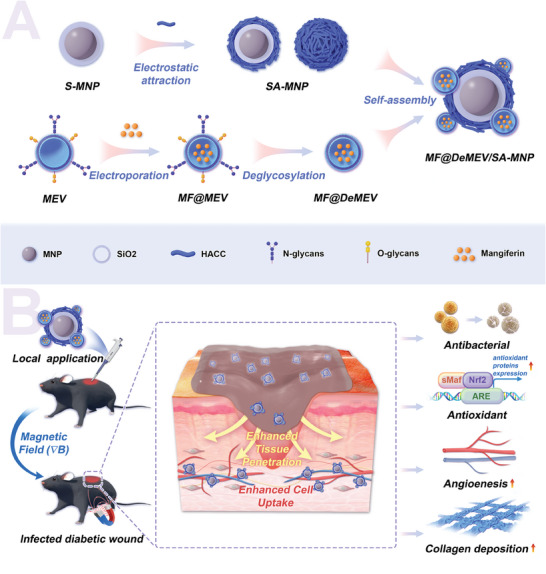
Schematic illustration showing A) the construction process of the glycoengineered EV‐based biohybrid nanorobot platform (MF@DeMEV/SA‐MNP) and B) its synergistic function for the therapy of infected diabetic wounds through dual‐enhanced cell and tissue penetration and multistep intervention strategy.

Herein, we present a glycoengineered EV‐based biohybrid nanorobot system (MF@DeMEV/SA‐MNP) capable of dual‐enhanced cell and tissue penetration, as well as multiple biological effects, for diabetic wound repair (**Figure** **1**). The artificial nanorobot (SA‐MNP) with Fe_3_O_4_ magnetic nanoparticle (MNP) as the “core” and SiO_2_ as the “shell”, was surface modified with antibacterial 2‐hydroxypropyltrimethyl ammonium chloride chitosan (HACC), and functioned as the synthetic unit of our biohybrid system. As the actuation source, external magnetic fields (∇*B*, the use of the same symbol for external magnetic field and gradient magnetic field) endowed the nanorobot with movement ability and notably enhanced tissue penetration of the entire system in wound sites. For the biological element, we developed a two‐step engineered EV (MF@DeMEV), in which milk‐derived extracellular vesicles (MEVs) were first loaded with mangiferin (MF), an antioxidant polyphenolic compound, and then the surface N‐ and O‐glycans were removed. Through glycoengineering, our nanoplatform greatly improved endothelial cell and fibroblast uptake efficiency and promoted the bioavailability of MF, thus reversing cell functions impaired by oxidative damage in vitro. In a mouse model of infected diabetic wounds, the dual‐enhanced penetration ability, along with antibacterial and antioxidant properties, synergistically accelerated wound healing by ameliorating re‐epithelialization, collagen deposition, and angiogenesis. To the best of our knowledge, this is the first study that exploits all‐in‐one biohybrid nanorobot platform for wound repair by integrating magnetically actuated nanorobots with engineered EVs, providing new insights into the fields of biomaterials and nanomedicine. In addition, this strategy can be applied to other diseases because of its programmability to customize functions according to different pathological environments through various EV engineering and versatile MNRs designs.

## 2 Results

### 2.1 Preparation, Characterization, and Magnetic Actuation of MF@DeMEV/SA‐MNPs

**Figure 2:**
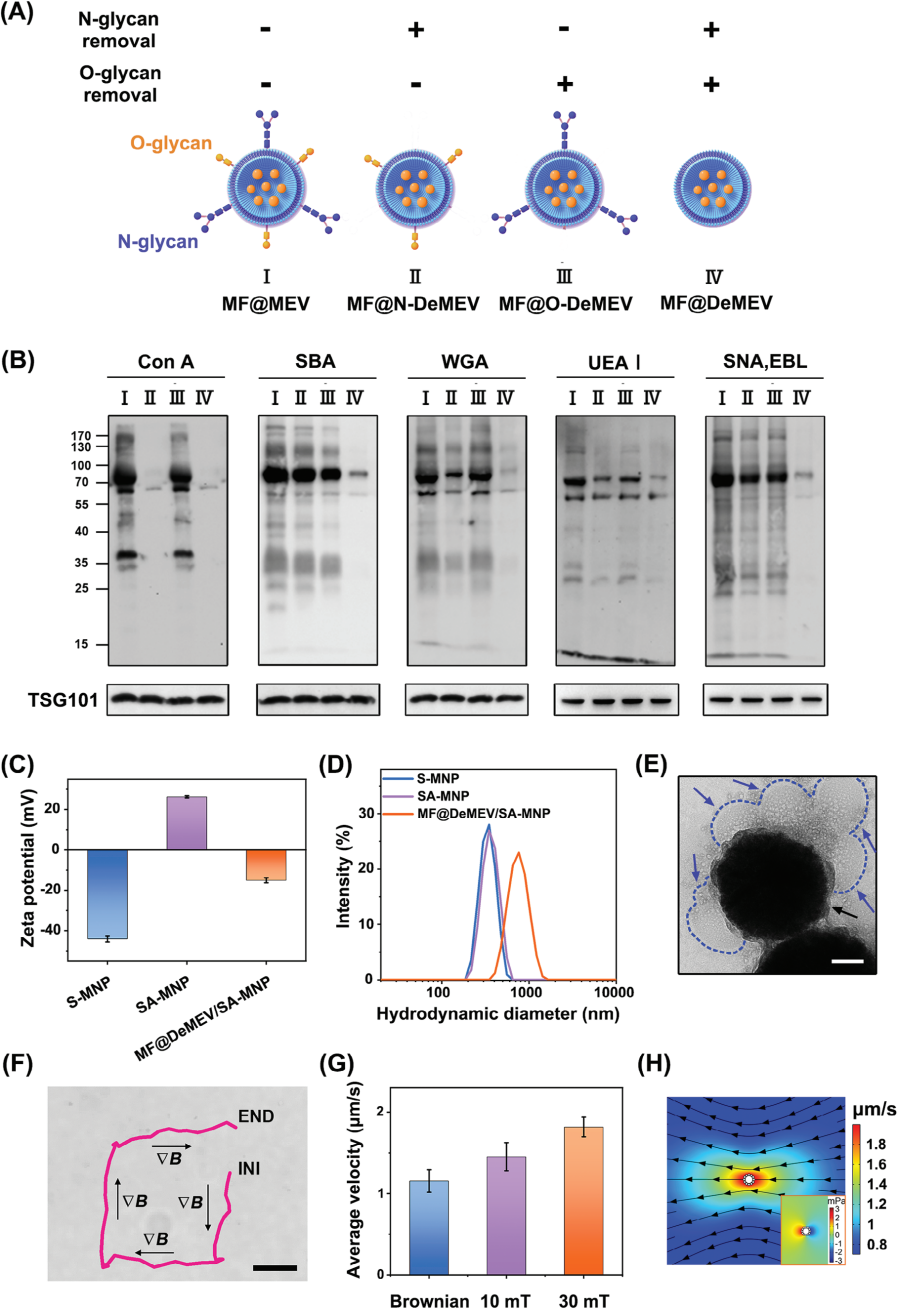
Preparation, characterization, and magnetic actuation of MF@DeMEV/SA‐MNPs. A) Schematic illustration of the construction of three types of glycoengineered MF@MEVs. B) Lectin blot analysis identified altered glycosylation profiles of MF@MEVs after deglycosylation enzyme treatment. MF@MEV was not treated by enzymes was used as a control. TSG101 was used as the reference gene. Lectins used for detections and their specificities of glycosylation structures are listed in Table 1. C,D) Zeta potential C) and size distribution D) of S‐MNP, SA‐MNP, and MF@DeMEV/SA‐MNP. E) TEM image of MF@DeMEV/SA‐MNP. Blue and black arrows point to MF@DeMEVs and SA‐MNP, respectively. Scale bar: 100 nm. F) Trajectory of individual MF@DeMEV/SA‐MNP under the local magnetic field generated by a magnet within 50 s. Scale bar: 20 µm. G) Mean velocities of MF@DeMEV/SA‐MNPs driven by different gradient magnetic fields (measured at the nanorobot's site). H) Numerical simulation of fluid flow and fluid pressure (inset) around a MF@DeMEV/SA‐MNP moving to the left under a gradient magnetic field.

To improve the bioavailability of MF and maximize its antioxidant capacity, we used an electroporation approach to load MF into MEVs, which we named MF@MEV. The encapsulation efficiency (EE) of electroporation for loading MF was 37.43 ± 1.67%. Our preliminary experiments showed that oxidative stress damage impaired the endothelial cell and fibroblast uptake of MF@MEVs (refer to 2.3. The “Dual‐Enhanced Penetration” Ability of MF@DeMEV/SA‐MNPs for details). The decreased uptake efficiency of EVs is usually accompanied by weakened therapeutic effects.^[^ ^19^ ^]^ To solve this problem, we removed N‐ and/or O‐glycans, which have been reported to inhibit the cellular uptake of EVs,^[^ ^20^, ^21^ ^]^ from MF@MEVs. The three types of glycoengineered MF@MEVs were named MF@N‐DeMEV, MF@O‐DeMEV, and MF@DeMEV (**Figure** **2**A). Five lectins were used to detect the glycosylation profiles (**Table** **1**), and the lectin blot assay confirmed that the N‐ and/or O‐linked glycans were successfully removed (Figure 2B). Our team has been investigating hydrogels for many years and has exploited several photo‐crosslinking hydrogel platforms, which have made outstanding contributions to the healing of skin and oral wounds.^[^ ^22^, ^23^, ^24^ ^]^ However, these platforms lack movement ability and cannot deliver EVs to deep and surrounding wound tissue. Therefore, we constructed different biohybrid nanorobots by combining four MEVs with SA‐MNPs using a simple electrostatic attraction method. Cellular uptake assays showed that the damaged cellular uptake of the biohybrid nanorobots was reversed only when N‐ and O‐ glycans were simultaneously deprived (refer to 2.3. The “Dual‐Enhanced Penetration” Ability of MF@DeMEV/SA‐MNPs for details). Therefore, the MF@DeMEVs and MF@DeMEV/SA‐MNPs were selected for further studies. Several assays were used to characterize the MF@DeMEVs. Transmission electron microscopy (TEM) observed that MEVs and MF@DeMEVs had similar inwardly concave cup‐like structures (Figure S1A, Supporting Information). Western blot analysis revealed that EV‐specific markers, including TSG101, CD9, and CD81, were highly expressed in both types of EVs (Figure S1B, Supporting Information). Next, as measured by nanoparticle tracking analysis (NTA), the mean size of the two EVs was 155.86 ± 1.85 and 155.47 ± 0.59 nm in diameter, respectively (Figure S1C,D, Supporting Information). These data indicated that MF loading and deglycosylation of MEVs had almost no influence on the morphology, surface markers, or particle size of the MEVs.

**Table 1:** Lectins utilized for detection of EV glycosylation patterns.

Lectin	Common Abbreviation	Primary Sugar Specificity
Concanavalin A	Con A	Mannose, Glucose
Soybean agglutinin	SBA	Galactose, N‐Acetylgalactosamine
Wheat Germ agglutinin	WGA	N‐Acetylglucosamine
*Ulex Europaeus* agglutinin I	UEA I	Fucose, Arabinose
Sambucus Nigra Lectin	SNA, EBL	Sialic Acid

Subsequently, S‐MNPs, SA‐MNPs, and MF@DeMEV/SA‐MNPs were characterized. The X‐ray diffraction (XRD) spectrum indicated the crystalline phase of Fe_3_O_4_ in S‐MNPs (Figure S2A, Supporting Information). The fourier transform infrared reflection (FTIR) spectra indicated the presence of Fe─O, Si─O─Si and N─H bonds in S‐MNPs and SA‐MNPs (Figure S2B, Supporting Information). Because of the large number of Si─O bonds on the surface of the S‐MNPs, the S‐MNPs exhibited a negative zeta potential (Figure 2C). When the S‐MNPs and HACC were mixed in an aqueous solution, HACC was adsorbed on the surface of the S‐MNPs because of the mutual attraction of static electricity, causing the SA‐MNPs to become positively charged. The MF@DeMEV/SA‐MNPs exhibited a negative zeta potential after the negatively charged MF@DeMEVs were adsorbed onto the surface of the positively charged SA‐MNPs. As measured by dynamic light scattering (DLS) (Figure 2D), the particle size of SA‐MNPs was 424.4 nm, which was slightly larger than that of the S‐MNPs (417.8 nm), and the diameter of MF@DeMEV/SA‐MNPs was 778.3 nm. The TEM images in Figure S2C,D (Supporting Information) showed that a thin layer of SiO_2_ existed on the surface of S‐MNPs and SA‐MNPs, while HACC could not be seen. The mean diameters of the two species were 378.9 ± 25.9 and 380.6 ± 31.1 nm, respectively. As shown in Figure 2E, the MF@DeMEVs were wrapped around the surface of the SA‐MNPs, resulting in an average diameter of 694.2 ± 16.2 nm. In general, due to the effect of solvent expansion and the existence of electric double layer, the particle size in water is larger than that in dry state. Moreover, HACC cannot be seen in TEM images but shows a stretching state in aqueous solution. These explain why the sizes of the three particles detected by DLS were slightly larger than those detected by TEM. Then, the drug release behavior of MF@DeMEV/SA‐MNPs was investigated. As shown in Figure S3 (Supporting Information), the exposure to external magnetic fields (∇*B*) accelerated the release of MF in the first 12 h, but overall, there was no difference in the release percent. After 72 h, 86.77 ± 2.42% and 88.19 ± 2.10% of MF was released from MF@DeMEV/SA‐MNPs with or without ∇*B*, and no sudden release was observed in both cases. Taken together, we presented strong proof that our biohybrid nanorobot platform was successfully constructed, and it had relatively sustained drug release to improve the bioavailability of MF.

To explore the transformation and locomotion of MF@DeMEV/SA‐MNPs in external magnetic fields, gradient magnetic fields (∇*B*) of controllable strength and direction generated by a magnet were applied to the nanorobots. To be specific, the sample cell was placed in the local magnetic field perpendicular to the magnet (Figure S4A, Supporting Information). The magnet generated a gradient field to drag and align the MF@DeMEV/SA‐MNPs with the field. When the direction of the external magnetic field changed by changing the alignment of the magnet, the moving direction of the MF@DeMEV/SA‐MNP altered, inducing a quadrate trajectory in 50 s (Figure 2F; Movie S1, Supporting Information). While when external magnetic fields (∇*B*) were removed, only Brownian motion could be observed (Figure S4B and Movie S2, Supporting Information). In addition, the average velocities of the MF@DeMEV/SA‐MNPs were calculated, which indicated that the biohybrid nanorobots could respond to different magnetic field strengths (Figure 2G). However, as the magnetic field strength increased from 10 to 30 mT, the speed of the nanorobot increased by only 1.3 times (from 1.45 to 1.82 µm s^−1^), which may be due to the strong hydrodynamic resistance and weak magnetic drag force of the nanoparticles. Additionally, we found that the transformation of single MF@DeMEV/SA‐MNP to chain‐like swarm in external magnetic fields (∇*B*) could boost actuation performance. When the concentration of MF@DeMEV/SA‐MNPs was increased from 0.01 to 0.1 mg mL^−1^, the nanorobots aggregated into chain arrays, which segregated from each other, due to the magnetic dipole and hydrodynamic interactions among them. The velocity of the chain‐like swarm varied with its size when the gradient field strength was fixed, as confirmed by the different trajectory lengths shown in Figure S4C (Supporting Information). Moreover, the speed of the chain‐like nanorobot swarm increased with magnetic field strength, reaching a higher speed than that of individual nanorobot (Figure S4D and Movie S3, Supporting Information). In detail, for chains with average length of 44.2 µm, the speed could achieve 3.1 µm s^−1^ at *B* = 10 mT, which was 2 times that of single nanorobot. This increase in speed can be attributed to the enhanced magnetic force in the chain‐like swarm composed of many MF@DeMEV/SA‐MNPs. Therefore, using the concentration of MF@DeMEV/SA‐MNPs above 0.1 mg mL^−1^ could promote the magnetic actuation performance.

Furthermore, the fluid flow around the moving MF@DeMEV/SA‐MNP was analyzed via numerical simulation. As shown in Figure 2H, the nanorobot was set at a constant velocity of 2 µm s^−1^, which was used to simulate directional motion under a gradient magnetic field. The calculated fluid velocity around the robot exhibited a non‐uniform distribution owing to the geometric features of the biohybrid nanorobot. When the robot moved, positive and negative pressure fields were generated in the forward and backward directions with a difference of up to 6 mPa. As a result, the micro‐disturbances and mass transfer caused by the flow and pressure fields around the robot help the nanorobot platform cross obstacles in real organisms. These results indicated that MF@DeMEV/SA‐MNPs displayed controllability and efficiency in magnetic field actuated motion.

### 2.2 In Vitro Biocompatibility Evaluation

Good biocompatibility is a fundamental requirement of biomaterials. Therefore, the cytotoxicity of MF@DeMEV/SA‐MNPs was examined in endothelial cells and fibroblasts using the Cell Counting Kit‐8 (CCK‐8) assay. Endothelial cells and fibroblasts are two types of cells in the deep skin tissue that play important roles in wound healing. Briefly, endothelial cells participate in neovascularization, while fibroblasts act as the main components of granulation tissue to synthesize new extracellular matrix (ECM) and help contract wounds.^[^ ^25^ ^]^ As illustrated in Figure S5A–D (Supporting Information), 100 µg mL^−1^ of MF@DeMEV/SA‐MNPs had no effect on the survival of endothelial cells and fibroblasts for 48 or 72 h, showing excellent biosafety, while the cell viability decreased when the concentration reached 150 µg mL^−1^ in fibroblasts for 72 h. Thus, MF@DeMEV/SA‐MNPs with the concentration of 100 µg mL^−1^ was selected for the subsequent cell‐based experiments. Moreover, we investigated the cytotoxicity of MF@DeMEV/SA‐MNPs exposed to external magnetic fields (∇*B*). As shown in Figure S5E–H (Supporting Information), cell viability exceeded 90% in every group, suggesting that MF@DeMEV/SA‐MNPs had no negative effect on the viability of endothelial cells or fibroblasts in the presence of external magnetic fields (∇*B*). Next, we estimated the hemocompatibility of the MF@DeMEV/SA‐MNPs using a hemolysis test (Figure S6, Supporting Information). The supernatants from the four MF@DeMEV/SA‐MNP groups were similar to those from the negative control (PBS) group. The hemolysis ratios (HRs) were 0.29%, 1.40%, 1.62%, and 1.96% for the 50, 100, 150, and 200 µg mL^−1^ of MF@DeMEV/SA‐MNPs, respectively. Biomaterials with HR <5% are permissible.^[^ ^1^ ^]^ Taken together, our results demonstrated good biocompatibility of MF@DeMEV/SA‐MNPs in vitro.

### 2.3 The “Dual‐Enhanced Penetration” Ability of MF@DeMEV/SA‐MNPs

**Figure 3:**
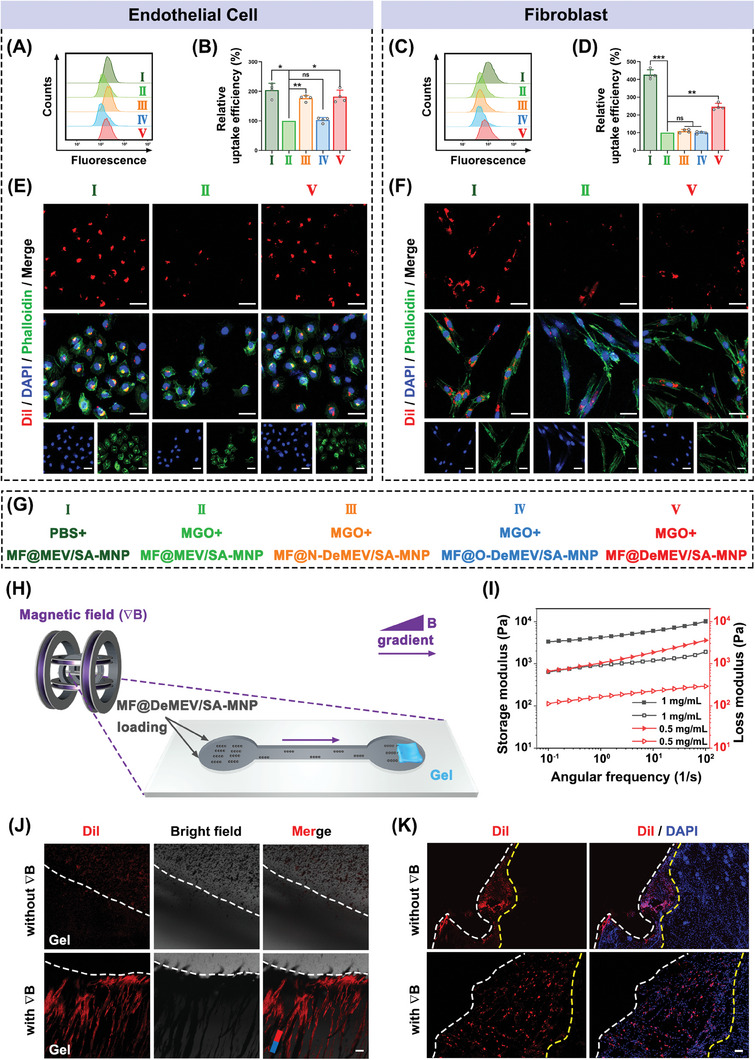
The “Dual‐Enhanced Penetration” ability of MF@DeMEV/SA‐MNPs. A–D) Cellular uptake efficiency of different types of nanorobots in endothelial cells A,B) and fibroblasts C,D) detected by flow cytometry (n = 4). E,F) CLSM images showed the cellular uptake situation of MF@MEV/SA‐MNPs or MF@DeMEV/SA‐MNPs in endothelial cells E) and fibroblasts F) in each group. Red, blue, and green fluorescence represented Dil‐labled MEVs, cellular nuclei, and cytoskeleton, respectively. Scale bar: 50 µm. G) The grouping information of A–F. H) Schematic illustration showing the 3D collagen gel model detecting the penetration ability of magnetically actuated MF@DeMEV/SA‐MNPs. I) Storage modulus and loss modulus of collagen gels crosslinked at different concentrations. J) CLSM images showed the penetration situations of MF@DeMEV/SA‐MNPs in 3D collagen gels with or without external magnetic field (∇*B*). White dotted lines represented the edges of collagen gels. Red fluorescence represented Dil‐labled MF@DeMEV/SA‐MNPs. Scale bar: 100 µm. K) Fluorescence microscope images showed the penetration situations of MF@DeMEV/SA‐MNPs in the wound dermis with or without external magnetic field (∇*B*). White dotted lines and yellow dotted lines represented the wound margins and the farthest penetration range, respectively. Red fluorescence represented Dil‐labled MF@DeMEV/SA‐MNPs. Scale bar: 100 µm. Data were presented as Mean ± SD; ns no significant, * p < 0.05, ** p < 0.01, *** p < 0.001.

Methylglyoxal (MGO) is one of advanced glycation end products (AGEs), and is an important factor that induces diabetic complications by causing oxidative stress.^[^ ^26^ ^]^ In this study, MGO was used to establish a cellular model of oxidative stress. The half‐maximal inhibitory concentration of MGO in endothelial cells and fibroblasts was 800 and 600 µM, respectively (Figure S7A,B, Supporting Information). In addition, MGO‐induced oxidative stress impaired the cellular uptake of MF@MEVs in a dose‐dependent manner (Figure S7C–F, Supporting Information). To reverse this damage, three glycoengineered MF@MEVs were established (Figure 2A,B) and synthesized with SA‐MNPs. Flow cytometry was used to evaluate the cellular uptake efficiency in the different groups. As shown in **Figure** **3**A,B, both MF@N‐DeMEV/SA‐MNPs and MF@DeMEV/SA‐MNPs restored cellular uptake to near‐normal levels in endothelial cells. While in fibroblasts, only the MF@DeMEV/SA‐MNPs reversed the impaired cellular uptake efficiency (Figure 3C,D). Therefore, MF@DeMEV/SA‐MNPs were selected for subsequent experiments, and confocal laser scanning microscopy (CLSM) further confirmed their ability to improve the cellular uptake efficiency (Figure 3E,F). The quantitative results of the CLSM showed a similar trend to those of the flow cytometry (Figure S8A,B, Supporting Information). To explore the mechanism by which MF@DeMEV/SA‐MNPs enhanced cellular uptake efficiency, we used different internalization inhibitors to pretreat the cells. In particular, simvastatin, wortmannin, filipin, and chlorpromazine inhibited lipid raft‐dependent endocytosis, macropinocytosis, caveolae‐dependent endocytosis, and clathrin‐dependent endocytosis, respectively. As shown in Figure S9A,C (Supporting Information), endothelial cells internalized MF@MEV/SA‐MNPs mainly through macropinocytosis and caveolae‐dependent endocytosis, while in fibroblasts, macropinocytosis and clathrin‐dependent endocytosis were the two main endocytic pathways. Interestingly, lipid raft‐dependent endocytosis and caveolae‐dependent endocytosis became one of the main pathways during the internalization of MF@DeMEV/SA‐MNP in endothelial cells and fibroblasts, respectively (Figure S9B,D, Supporting Information). These results indicated that our glycoengineered biohybrid nanorobots could enhance their uptake efficiency in endothelial cells and fibroblasts through changing endocytic pathways.

Although MF@DeMEV/SA‐MNPs exhibited high speed and controllability in aqueous environments, their penetration and sustained motion performance in biological tissue environments, such as the ECM, still needs to be studied, which is crucial for infiltrating the surrounding and deep tissue of wounds. Therefore, we performed magnetic penetration experiments on MF@DeMEV/SA‐MNPs using a 3D collagen gel model to simulate dermal tissue (Figure 3H). We prepared collagen gels of different moduli by varying the gel concentration. Considering that at the wound site, the dermis and hypodermis are impaired, leading to the exposure of connective tissue and exudates such as mucus, the group with elastic modulus of 650 pa (0.5 mg mL^−1^) was selected for subsequent experiments (Figure 3I). For the magnetic penetration experiment, the gel was placed on one side of the double‐well chamber, and Dil‐labeled MF@DeMEV/SA‐MNPs were added to the other side. The observation chamber was filled with PBS. After that, MF@DeMEV/SA‐MNPs were induced to move toward the gel by an applied gradient magnetic field (∇*B*, 10 mT) for 30 min. From the CLSM images and their corresponding quantitative analysis, it can be seen that static MF@DeMEV/SA‐MNPs hardly penetrated into the gel, while they could spontaneously form needle‐like swarm and enter into the deep part of the gel after an external magnetic field (∇*B*) was introduced (Figure 3J; Figure S8C, Supporting Information). The moving direction of MF@DeMEV/SA‐MNPs in a gel was also investigated. As shown in Figure S10 (Supporting Information), when external magnetic gradient was applied, the MF@DeMEV/SA‐MNPs moved along the gradient direction, generating needle‐like swarms along the magnetic induction line. These results indicated that the biohybrid nanoplatform exhibited outstanding penetration performance in a simulated dermal environment.

To detect the penetration capacity of the MF@DeMEV/SA‐MNPs into real wound tissue, we produced cross‐sectional frozen sections of the wound dermis and observed their distribution. Dil‐labeled MF@DeMEV/SA‐MNPs were applied to the mice's dorsal wounds with or without external magnetic fields (∇*B*) for 4 h, and then the wound tissue was dissected and prepared for imaging. As shown in Figure 3K; Figure S8D (Supporting Information), the fluorescent molecules were found to infiltrate in the dermis layer of wound tissue with the farthest penetration distance of 1266.5 ± 168.2 µm when our robots were under external magnetic fields (∇*B*), while the distance was only 362.1 ± 119.2 µm without external magnetic fields (∇*B*). These results indicated that our magnetic field‐driven nanorobots had excellent tissue penetration properties and could deliver MF‐loaded MEVs to the surrounding and deep wound tissue with greater efficiency. In summary, we demonstrated that MF@DeMEV/SA‐MNPs displayed a dual‐enhanced penetration ability and that they enhanced penetration at both the cell and tissue levels.

### 2.4 MF@DeMEV/SA‐MNPs Reversed Cell Functions Impaired by Oxidative Damage

**Figure 4:**
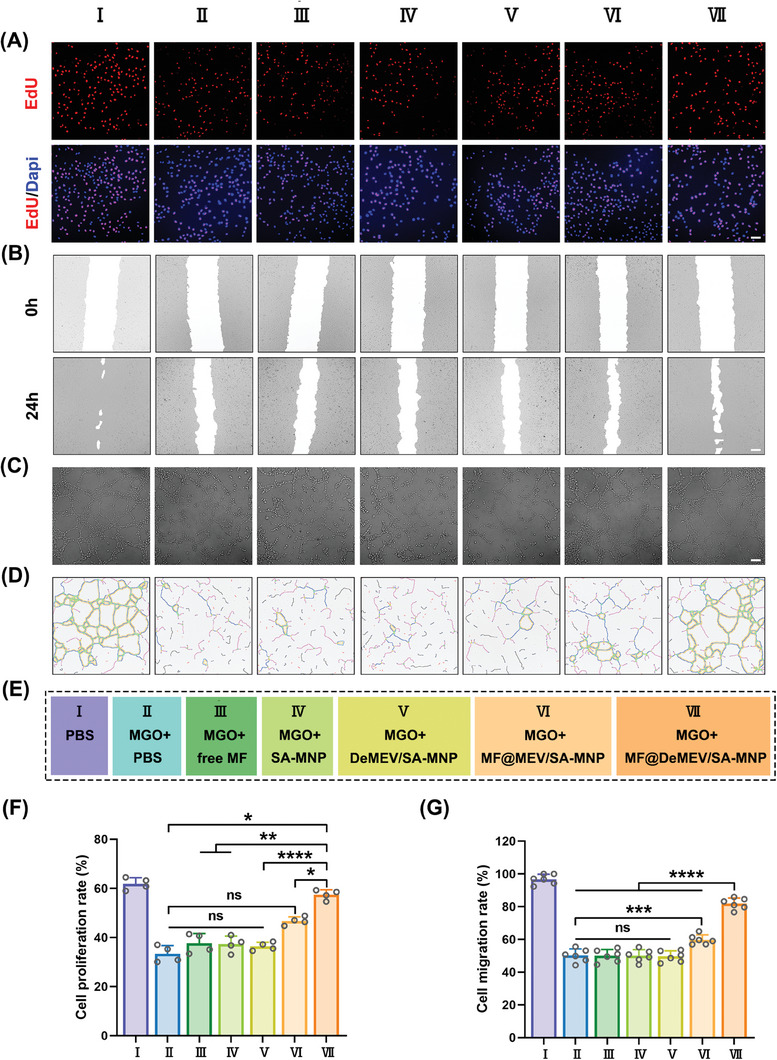
MF@DeMEV/SA‐MNPs reversed endothelial cell functions impaired by oxidative damage. A,F) The proliferation rate of endothelial cells after different treatments analyzed by EdU assay. Red and blue fluorescence represented proliferative cells and cellular nuclei, respectively (n = 4). Scale bar: 50 µm. B,G) The migration rate of endothelial cells after different treatments analyzed by wound healing assay (n = 6). Scale bar: 100 µm. C,D) Microscope images C) of tube formation of endothelial cells after different treatments, and their corresponding images processed by ImageJ D). Scale bar: 100 µm. E) The grouping information. Data were presented as Mean ± SD; ns no significant, * p < 0.05, ** p < 0.01, *** p < 0.001, **** p < 0.0001.

Damage due to oxidative stress is a key factor affecting the function of endothelial cells and fibroblasts, thus preventing diabetic wound healing. To investigate the functional effects of the MF@DeMEV/SA‐MNPs, we treated the cells damaged by MGO with PBS (group II), free MF (group III), SA‐MNPs (group IV), DeMEV/SA‐MNPs (group V), MF@MEV/SA‐MNPs (group VI), or MF@DeMEV/SA‐MNPs (group VII). Cells in group I were treated only with PBS and represented the normal cell group without MGO damage. We then observed the cell behavior using 5‐ethynyl‐2′‐deoxyuridine (EdU) assays, wound healing assays, and in vitro tube formation assays. First, as shown in **Figures** **4**A,F and S11A,C (Supporting Information), EdU assays were performed to assess the proliferation ability of the two cells. Consistent with our expectations, the proliferation rate of the cells treated with MF@DeMEV/SA‐MNPs increased the most. MF@MEV/SA‐MNPs without deglycosylation reversed the cellular proliferation rate to a certain extent; however, the rate was lower than that of MF@DeMEV/SA‐MNPs. While free MF without delivery system, SA‐MNPs without two‐step engineered MEVs, or DeMEV/SA‐MNPs without MF loaded had little effect on the improvement in cellular proliferation ability. Next, we evaluated the cellular migration ability by conducting in vitro wound healing assays, and the results showed a similar trend to those of the EdU assays (Figure 4B,G; Figure S11B,D, Supporting Information). The angiogenic ability of endothelial cells was detected using a vascular network formation assay in Matrigel. We used the total tube length, number of nodes, and number of master segments as the three indicators to evaluate their ability. As shown in Figure 4C,D; Figure S12 (Supporting Information) free MF, SA‐MNPs, or DeMEV/SA‐MNPs did not reverse the injured angiogenic ability of endothelial cells. In contrast, MF@MEV/SA‐MNPs and MF@DeMEV/SA‐MNPs enhanced this ability to various degrees, with MF@DeMEV/SA‐MNPs exhibiting the most positive effect. The above evidence strongly suggested that our biohybrid nanorobots greatly reversed the endothelial cell and fibroblast functions that were impaired by oxidative stress.

### 2.5 MF@DeMEV/SA‐MNPs Alleviated Oxidative Stress via Activating the Nrf2 Signaling Pathway

**Figure 5:**
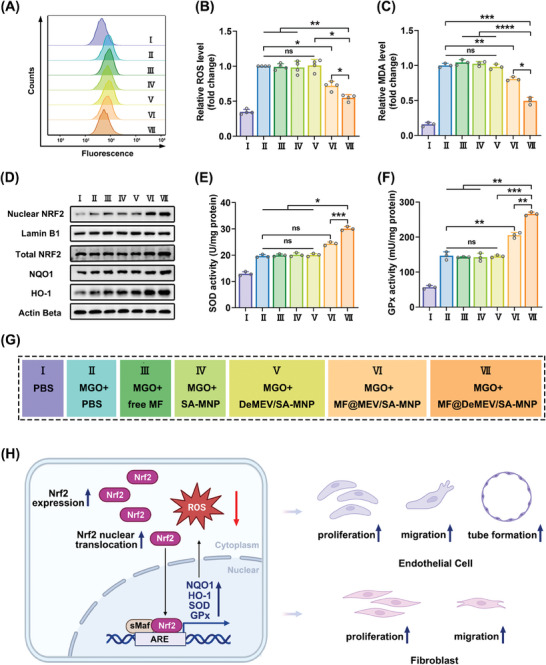
MF@DeMEV/SA‐MNPs alleviated oxidative stress via activating the Nrf2 signaling pathway in endothelial cells. A,B) Intracellular ROS levels in endothelial cells after different treatments measured by flow cytometry (n = 4). C) MDA levels in endothelial cells after different treatments (n = 3). D) Western blotting analysis of nuclear NRF2, total NRF2, NQO1 and HO‐1 in endothelial cells after different treatments. Lamin B1 and Actin Beta were used as the reference genes. E) SOD activities in endothelial cells after different treatments (n = 3). F) GPx activities in endothelial cells after different treatments (n = 3). G) The grouping information. H) Schematic illustration showing the cascading action mechanism of MF@DeMEV/SA‐MNPs in endothelial cells and fibroblasts. Data were presented as Mean ± SD; ns no significant, * p < 0.05, ** p < 0.01, *** p < 0.001, **** p < 0.0001.

Subsequently, we explored the antioxidant activity of MF@DeMEV/SA‐MNPs. ROS are recognized indicators for evaluating the levels of oxidative stress. Thus, we first measured the intracellular ROS levels using the 2′,7′‐Dichlorodihydrofluorescein Diacetate (DCFH‐DA) approach (**Figure** **5**A,B; Figure S13A,B, Supporting Information). Fluorescence intensity was detected by flow cytometry, and the results suggested that MF@DeMEV/SA‐MNPs reduced elevated ROS levels by approximately half. Although MF@MEV/SA‐MNPs also suppressed ROS generation, the effect was not as strong as that of MF@DeMEV/SA‐MNPs. Lipid peroxidation, a well‐known cellular damage mechanism, is typically induced by the overproduction of intracellular ROS. Malondialdehyde (MDA) is a byproduct of this process, and its measurement provides an accurate index of lipid peroxidation.^[^ ^27^ ^]^ In order to assess the cytoprotective effect of our robots on intracellular lipid peroxidation, we measured MDA levels in endothelial cells and fibroblasts from different treatment groups. As demonstrated in Figures 5C and S13C (Supporting Information), MF@DeMEV/SA‐MNPs exhibited a stronger inhibitory effect on lipid peroxidation than MF@MEV/SA‐MNPs, while MDA levels in the other groups did not decrease. Overall, we provided evidence that our nanorobot system significantly alleviated MGO‐induced oxidative stress in endothelial cells and fibroblasts.

MF has been shown to possess antioxidant activity via the Nrf2‐mediated antioxidant system.^[^ ^28^ ^]^ To test whether MF@DeMEV/SA‐MNPs could deliver MF efficiently and exert this function, we measured the protein expression levels of total and nuclear Nrf2, along with NAD(P)H: quinone oxidoreductase 1 (NQO1) and heme oxygenase‐1 (HO‐1), in differently treated endothelial cells and fibroblasts (Figure 5D; Figures S13D and S14, Supporting Information). The activities of superoxide dismutase (SOD) and glutathione peroxidase (GPx) were also measured (Figure 5E,F; Figure S13E,F, Supporting Information). NQO1, HO‐1, SOD, and GPx are four Nrf2‐regulated endogenous antioxidant enzymes. Western blot assays showed that stimulation with MGO followed by treatment with PBS, free MF, SA‐MNPs, or DeMEV/SA‐MNPs had no effect on the protein level of total Nrf2 compared to the normal cell group, but interestingly, increased the nuclear Nrf2 level. This phenomenon can be explained by the fact that Nrf2 tends to transfer from the cytoplasm to the nucleus under stress conditions such as oxidative stress.^[^ ^2^, ^29^ ^]^ Meanwhile, MF@MEV/SA‐MNPs and MF@DeMEV/SA‐MNPs increased the protein levels of both total and nuclear Nrf2, with MF@DeMEV/SA‐MNPs having greater promoting effect. Consistent with the above findings, the levels of NQO1 and HO‐1, as well as the activities of SOD and GPx, were elevated in the PBS, free MF, SA‐MNP, DeMEV/SA‐MNP, MF@MEV/SA‐MNP, and MF@DeMEV/SA‐MNPs groups to deal with oxidative stress, with MF@DeMEV/SA‐MNPs showing the greatest promoting effect.

In summary, the cell experiments together demonstrated that the more effective delivery of MF due to the promoted cellular uptake of MF@DeMEV/SA‐MNPs could relieve oxidative stress and thus reverse damaged endothelial cell and fibroblast functions by enhancing Nrf2 expression levels in whole cells, facilitating its nuclear transfer, and activating downstream antioxidant enzymes (Figure 5H).

### 2.6 In Vitro Antibacterial Evaluation

HACC is a chitosan derivative that kills bacteria by binding quaternary ammonium groups to their cytoplasmic membranes of bacterial cells.^[^ ^30^ ^]^ To verify its antibacterial properties, we conducted a series of experiments. First, our results of the plate count method illustrated that S‐MNPs not modified with HACC had no antibacterial activity, while the bacterial survival rate of *Staphylococcus aureus* in SA‐MNPs and MF@DeMEV/SA‐MNPs groups was only 15.13 ± 2.58% and 20.31 ± 0.91% respectively (Figure S15A,B, Supporting Information), and the rate of *Escherichia coli* in the two groups was only 7.69 ± 1.4% and 21.83 ± 3.37% respectively (Figure S15E,F, Supporting Information). Interestingly, although there was no statistically significant difference, the SA‐MNPs exhibited a slightly better antibacterial effect than the MF@DeMEV/SA‐MNPs at the same concentration. This may be because the combination of MF@DeMEVs and SA‐MNPs partially covered the polymer cationic groups of HACC. Subsequently, a live/dead bacterial staining assay was performed. In this assay, Propidium Iodide (PI) dye can only penetrate dead bacteria through damaged cell membranes and emits red fluorescence, while 4′,6diamidino‐2‐phenylindole (DAPI) binds to the DNA of both live and dead bacteria and emits blue fluorescence. As illustrated in Figure S15C,D,G,H (Supporting Information), the bacterial death rates of *S. aureus* and *E. coli* in SA‐MNPs and MF@DeMEV/SA‐MNPs groups were all over 65%. Overall, these results strongly demonstrated that our HACC‐modified biohybrid nanorobots possessed excellent antibacterial capabilities.

An increasing number of studies have demonstrated the great potential of MNRs in antibiofilm applications.^[^ ^31^ ^]^ In order to examine the performance of our robots in destroying biofilms, crystal violet‐stained biofilm assay was conducted (Figure S16, Supporting Information). Consistent with the plate count method, the static S‐MNPs did not eliminate bacterial biofilms. In contrast, when we placed S‐MNPs in external magnetic fields (∇*B*), biofilm destruction could be observed, indicating their physical disruption effect on biofilms. In addition, we observed a significant antibiofilm effect in groups treated with static SA‐MNPs or MF@DeMEV/SA‐MNPs, both of which reduced *S. aureus* and *E. coli* biofilm residues by more than half. While SA‐MNPs or MF@DeMEV/SA‐MNPs under external magnetic fields (∇*B*) displayed the most effective antibiofilm abilities. This evidence demonstrated that our dynamic nanorobots could eliminate biofilms with high efficiency, which was attributed to the synergistic effects of HACC and physical disruption.

### 2.7 Treatment Effect of MF@DeMEV/SA‐MNPs on Infected Diabetic Wounds

**Figure 6:**
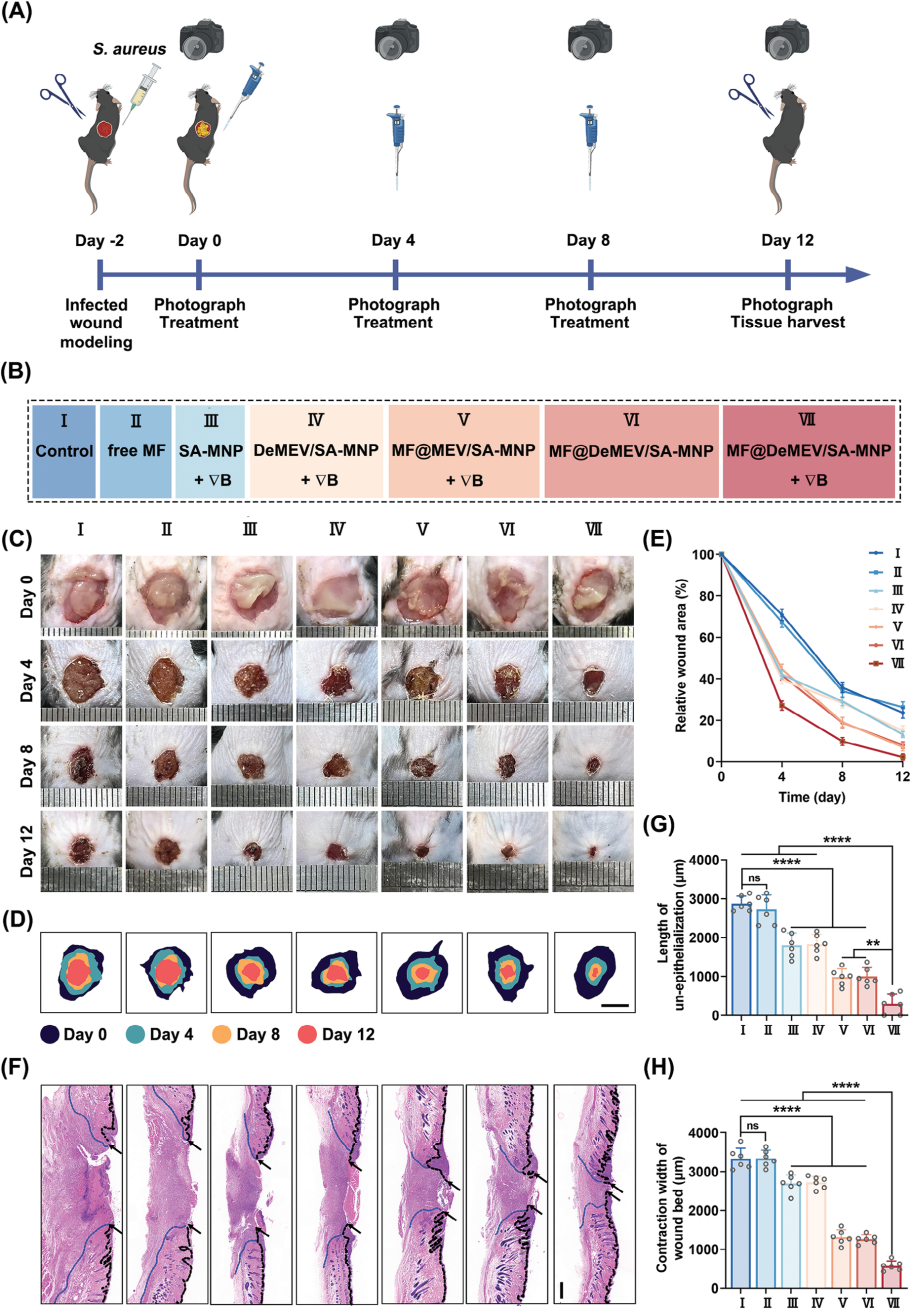
Treatment effect of MF@DeMEV/SA‐MNPs on infected diabetic wounds A) Schematic illustration showing the modeling and treatment procedures of *S. aureus*‐infected wounds in diabetic mice. B) The grouping information of the in vivo experiment. C) Representative images of wounds in each group at different time points. D) Schematic diagram of the wound healing process in each group during the 12 days. Scale bar: 5 mm. E) Quantitative analysis of the wound area in each group at different time points (n = 6). F) H&E staining of wound sections in each group on day 12 post‐treatment. Black arrows, black dotted lines, and blue dotted lines represented the edges of epithelialization, the boundaries of epidermis and dermis, and the edges of wound beds, respectively. Scale bar: 500 µm. G) Quantification analysis of the length of un‐epithelialization in F (n = 6). H) Quantification analysis of the contraction width of wound bed in F (n = 6). Data were presented as Mean ± SD; ns no significant, ** p < 0.01, *** p < 0.001, **** p < 0.0001.

To explore the potential of MF@DeMEV/SA‐MNPs for clinical treatment, we established *S. aureus* infected wound models in the dorsum of diabetic mice, and treated the wounds with PBS (control group), free MF, dynamic SA‐MNPs, dynamic DeMEV/SA‐MNPs, dynamic MF@MEV/SA‐MNPs, static MF@DeMEV/SA‐MNPs, and dynamic MF@DeMEV/SA‐MNPs on days 0, 4, and 8 (**Figure** **6**A,B). Because the mouse was kept in a 16 cm × 10 cm cage with one magnet on each side of the cage, the strength of magnetic field was 4 – 10 mT. The duration of the magnetic field was 12 h after each treatment. The wound conditions were recorded on days 0, 4, 8, and 12 (Figure 6C,D). Quantitative analysis revealed that PBS and free MF had no therapeutic effects, whereas other treatments promoted wound healing to varying degrees (Figure 6E; Figure S17, Supporting Information). As we expected, dynamic MF@DeMEV/SA‐MNPs exhibited the best curative effect throughout the treatment process, and the wounds in the group were almost healed on day 12. While the unclosed wound rate in static MF@DeMEV/SA‐MNP group was 8.05 ± 1.57%, suggesting that the external magnetic fields (∇*B*), which driven the biohybrid nanorobots to penetrate into the wounds, had great positive effect on promoting wound healing. In addition, the rates in the dynamic MF@MEV/SA‐MNP and static MF@DeMEV/SA‐MNP groups were lower than those in the dynamic SA‐MNP and dynamic DeMEV/SA‐MNP groups from day 8 onwards, emphasizing the importance of the synergy between MF and HACC.

**Figure 7:**
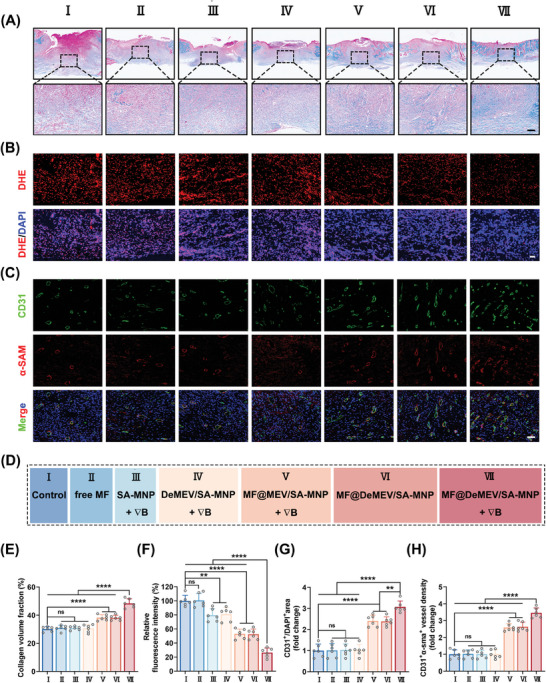
Masson's trichrome staining and fluorescence staining analysis of wound sections. A,E) Masson's trichrome staining of wound sections in each group for evaluating collagen deposition on day 12 post‐treatment (n = 6). Scale bar: 100 µm. B,F) Dihydroethidium (DHE) staining of wound sections in each group for evaluating ROS levels in wound tissue on day 12 post‐treatment (n = 6). Scale bar: 50 µm. C,G,H) Immunofluorescence staining of CD31 and α‐SMA of wound sections in each group for evaluating the angiogenesis in wound tissue on day 12 post‐treatment (n = 6). Scale bar: 50 µm. D) The grouping information. Data were presented as Mean ± SD; ns no significant, ** p < 0.01, **** p < 0.0001.

Next, histological analysis was performed on day 12 post‐modeling to investigate the degree of wound repair and regeneration in the different treatment groups. First, hematoxylin and eosin (H&E) staining was used to analyze the length of the epithelial tissue gap and the width of the wound bed, which are two typical indicators of wound healing (Figure 6F–H). The results displayed a trend similar to that of the wound healing rates. Briefly, the dynamic MF@DeMEV/SA‐MNP treated group exhibited the shortest epithelial gap and wound bed width, indicating the best wound‐healing conditions in the group. Although dynamic MF@MEV/SA‐MNPs and static MF@DeMEV/SA‐MNPs promoted the healing of infected diabetic wounds to a large extent, the effect was not as strong as that of dynamic MF@DeMEV/SA‐MNPs. Masson's trichrome staining was used to explore collagen synthesis and deposition levels during wound healing (**Figure** **7**A,E). Collagen deposition was promoted in the dynamic MF@MEV/SA‐MNP, static MF@DeMEV/SA‐MNP, and dynamic MF@DeMEV/SA‐MNP groups, whereas collagen deposition in the dynamic MF@DeMEV/SA‐MNP group was the densest and most regular. In contrast, no improvement in collagen deposition was observed in the PBS, free MF, dynamic SA‐MNP, and dynamic DeMEV/SA‐MNP groups.

Encouraged by the efficient oxidative stress‐alleviating ability of our biohybrid nanorobots in vitro, we investigated whether the nanoplatform could scavenge ROS in vivo. Therefore, we examined ROS levels at the wound site on day 12 using dihydroethidium (DHE) staining. As expected, a stark decline in ROS levels was observed after treatment with dynamic MF@DeMEV/SA‐MNPs (Figure 7B,F), indicating their excellent ROS‐scavenging performance in vivo. Dynamic SA‐MNPs, dynamic DeMEV/SA‐MNPs, dynamic MF@MEV/SA‐MNPs, and static MF@DeMEV/SA‐MNPs also relieved oxidative stress; however, the effect was not as good as that of dynamic MF@DeMEV/SA‐MNPs.

Then, immunofluorescence staining for CD31 and α‐smooth muscle actin (α‐SMA), biomarkers of endothelial cells and vascular smooth muscle cells, were utilized to evaluate the vascularization in wound bed. In detail, CD31 positive endothelial cells represented newly formed blood vessels (Figure 7C,G), while CD31 and α‐SMA double positive vessels represented mature vessels (Figure 7C,H). We found that compared with PBS, free MF, dynamic SA‐MNPs, and dynamic DeMEV/SA‐MNPs, which had little promoting effect on angiogenesis, dynamic MF@MEV/SA‐MNPs, static MF@DeMEV/SA‐MNPs, and dynamic MF@DeMEV/SA‐MNPs significantly improved vascular network formation. Notably, the highest vascular density was observed in the dynamic MF@DeMEV/SA‐MNP group, indicating that this group had the strongest pro‐angiogenic effect.

To confirm the antibacterial activity of MF@DeMEV/SA‐MNPs in vivo, we collected the wound fluid of mice in each group and analyzed the number of bacteria using plate count method on day 4 post‐treatment. As shown in Figure S18A,D (Supporting Information), except for free MF group, other groups exhibited good antibacterial effect. Specifically, dynamic SA‐MNP, dynamic DeMEV/SA‐MNP, dynamic MF@MEV/SA‐MNP, and dynamic MF@DeMEV/SA‐MNP groups had the similar antibacterial effect, and the bacterial survival rate was 35.39 ± 5.49%, 35.26 ± 5.99%, 34.72 ± 5.07%, 35.31 ± 5.47%, respectively. While the effect in static MF@DeMEV/SA‐MNP group was not good as that in dynamic MF@DeMEV/SA‐MNP group, and the rate was 47.46 ± 6.37%. The antibacterial effect of each group showed a similar trend in Gram staining analysis of wound sections (Figure S18B,E, Supporting Information). Overall, these results demonstrated that due to the synergistic effects of HACC and physical disruption, MF@DeMEV/SA‐MNPs possessed great antibacterial activity in vivo.

Finally, we estimated the biocompatibility of MF@DeMEV/SA‐MNPs in vivo. The main organs (heart, liver, spleen, lungs, and kidneys) were collected from diabetic mice with infected wounds 12 days after treatment, and H&E staining was performed. As shown in Figure S19 (Supporting Information), no significant tissue damage or obvious inflammatory lesions were observed in the different treatment groups, indicating that these nanorobots exhibited good biocompatibility in vivo.

Taken together, these results strongly proved that our biohybrid nanoplatform exhibited an outstanding treatment effect on infected diabetic wounds, which was attributed to its synergistic functions of dual‐enhanced penetration, antibacterial, and antioxidant abilities.

## 3 Discussion

The last decade has witnessed significant progress and breakthroughs in the field of biohybrid MNRs comprising artificial MNRs and biological components. For the artificial MNRs, we selected external magnetic fields as the actuation source because of the unique advantages of this strategy, such as ease of operation and remote maneuverability.^[^ ^10^, ^32^ ^]^ As expected, our magnetically driven nanorobots with Fe_3_O_4_ nanoparticle‐based core/shell structures exhibited toxic fuel‐free and non‐invasive characteristics, high temporal and spatial control, and specific navigational and reconfigurable abilities. Owing to these features, the biohybrid system exhibited excellent penetration ability and improved cargo retention in both the 3D collagen gel model and real wound sites. It is very important to develop this kind of active delivery system with high tissue penetration because the microenvironment of diabetic wounds is complicated, the tissue architecture is perturbed, the cell‐matrix interaction is unbalanced, and the bacteria can easily colonize. This causes the therapeutic agents of passive diffusion to be blocked by blood clots, tissue exudates, and biofilms, making it difficult to act in the dermis and even subcutaneous tissue of wounds.^[^ ^5^, ^25^, ^33^ ^]^ One research has established a microalgae‐based biohybrid microrobot system to penetrate blood clots and accelerate diabetic wound healing.^[^ ^5^ ^]^ Compared with this research, our present study went a step further and achieved enhanced penetration at the cellular level through introducing glycoengineered EVs as biological components.

In existing studies, the biological components of biohybrid magnetic MNRs are usually non‐motile pollen, spores, microalgae, active locomotive cells with flagella, such as sperm and bacteria, cell membranes/vesicles with high biocompatibility, and immune cells possessing phagocytic ability.^[^ ^34^, ^35^, ^36^, ^37^, ^38^, ^39^ ^]^ Here, we introduced an innovative concept in which two‐step engineered EVs functioned as biological elements and were integrated with the above‐mentioned nanorobots to construct a multifunctional biohybrid nanoplatform. Although some achievements have been made in isolating EVs from cell culture supernatants for drug delivery, the yield is so low that it cannot meet the requirements of large‐scale clinical applications.^[^ ^40^ ^]^ In view of the current limitations, researchers have recognized bovine milk as a more promising source of EVs, since bovine milk is abundant in nature and large quantities of EVs can be harvested from it on an industrial scale. Indeed, MEVs have been reported to be promising drug delivery systems owing to their high biocompatibility, safety, and accessibility, and many studies have successfully encapsulated medicinal small molecules, nucleic acids, and even peptide/protein drugs in MEVs for therapeutic applications.^[^ ^41^, ^42^, ^43^ ^]^ MF is a good candidate for treating diabetic wounds because of its antioxidant activity. However, its low solubility and poor transmembrane permeability limit its application.^[^ ^44^ ^]^ Therefore, we loaded MF into MEVs to improve its bioavailability and pharmacological activity. As we can see from the results, both natural and deglycosylated MEV delivery preserved the antioxidant activity of MF, with the latter having the better protective effect.

Two other studies have also discussed the healing effect of MF in diabetic wounds.^[^ ^45^, ^46^ ^]^ Wu et al. revealed the angiogenic activity of MF in the healing process, and attributed this effect to up‐regulated expression of VEGF and HIF‐1α, two important factors related to angiogenesis.^[^ ^45^ ^]^ We demonstrated that MF exerted antioxidant properties by activating the Nrf2‐antioxidant signaling pathway, which was consistent with the results found in the study by Lwin et al.^[^ ^46^ ^]^ The Nrf2 signaling pathway plays a crucial role in maintaining cellular redox homeostasis, and the activation of the pathway by MF has been reported to combat oxidative stress in arsenic‐induced lung injury, ovalbumin‐caused allergic rhinitis, cardiac fibrosis, renal ischemia/reperfusion, Parkinson's disease, and other organ or tissue damage.^[^ ^47^, ^48^, ^49^, ^50^, ^51^ ^]^ In response to oxidative stress, Nrf2 transcriptionally regulates ROS‐detoxifying and antioxidant genes by transferring from the cytoplasm to the nucleus and binding to antioxidant response elements (ARE).^[^ ^2^, ^52^ ^]^ Indeed, we observed the promotion of Nrf2 nuclear translocation and the consequent increase of antioxidant enzymes in MGO‐induced oxidative damaged endothelial cells and fibroblasts. However, the limited antioxidant processes initiated by the cells themselves could not scavenge the abnormally produced ROS. The application of our biohybrid system resolved this problem by delivering MF with higher efficiency and further facilitating Nrf2 activation.

Analysis of glycans is much more complicated than that of proteins and lipids because of their structural complexity; thus, research on EV surface glycomes has been lagging behind.^[^ ^53^ ^]^ However, there is growing awareness of the importance of glycan patterns in the EV field, and some studies have investigated their roles in the uptake efficiency of EVs by recipient cells. Nishida‐Aoki et al. found that the removal of N‐and/or O‐glycans from breast cancer EVs enhanced their uptake by endothelial cells.^[^ ^20^ ^]^ These findings were partially contrary to those in the study by Yamamoto et al., where the removal of N‐glycans increased melanoma cell‐derived EV uptake by macrophages, whereas the removal of O‐glycans had little effect.^[^ ^21^ ^]^ We found that N‐glycan‐deprived or N‐ and O‐glycan‐deprived, but not O‐glycan‐deprived MEVs restored the cellular uptake efficiency of biohybrid robots in endothelial cells. While in fibroblasts, the uptake efficiency could be enhanced only when both N‐ and O‐glycans were removed. The results of our and other studies suggest that EV uptake levels depend not only on different surface glycan profiles in EVs from diverse sources but also on the types of recipient cells.^[^ ^15^, ^54^ ^]^ However, there are limited researches about the mechanism how recipient cells recognize and internalize EVs under the action of glycan profiles. Among these researches, sialic acids and HSPGs are the two kinds of glycans studied most. For example, surface‐expressed α2,3‐linked sialic acids in B cell‐derived EVs are reported to combine sialoadhesin (CD169; Siglec‐1) in splenic marginal zone macrophages and mediate cellular uptake.^[^ ^55^ ^]^ In the current study, the changes in endocytic pathways preliminarily explained enhanced cellular uptake efficiency after glycoengineering. However, in‐depth mechanism exploration involving receptor‐mediated and glycan‐dependent patterns is limited by the lagging state of glycomics research. Therefore, more researches need to be performed to explore the roles of different glycans in EV uptake. This will benefit the development of more glycoengineered EVs for efficient drug delivery applications.

## 4 Conclusion 

We constructed a novel biohybrid nanorobot platform to accelerate diabetic wound healing. A magnetically driven nanorobot with HACC surface modification and glycoengineered MEVs loaded with MF served as the artificial unit and biological components of the platform, respectively. The movement ability of the nanorobot and the deglycosylation of the MEV membranes enhanced the penetration of the platform at both the tissue and cell levels. Additionally, the introduction of HACC and MF endowed the platform with antibacterial and antioxidant properties, respectively. Collectively, these functions synergistically promoted the repair of diabetic wounds through multistage interventions. More importantly, the strategy of combining autonomous mobile MNRs and multistep‐engineered EVs provides a broader perspective for the design of next‐generation biohybrid MNR platforms that can possess various tailor‐made functions and can be generalized to other diseases.

## Conflict of Interest

The authors declare no conflict of interest.

## Supporting information

Supporting Information

Supplemental Movie 1

Supplemental Movie 2

Supplemental Movie 3

## Data Availability

The data that support the findings of this study are available from the corresponding author upon reasonable request.
