# Supplementary material for: Biohybrid Nanorobots Carrying Glycoengineered Extracellular Vesicles Promote Diabetic Wound Repair through Dual‐Enhanced Cell and Tissue Penetration
Source: Adv Sci (Weinh). 2024 Jun 18;11(31):2404456. doi: 10.1002/advs.202404456 (PMC11336935; doi:10.1002/advs.202404456)
Supplement: Supplementary file 1 — Supporting Information [file ADVS-11-2404456-s004.docx]

Supporting Information

Biohybrid Nanorobots Carrying Glycoengineered Extracellular Vesicles Promote Diabetic Wound Repair through Cell and Tissue Dual-Enhanced Penetration

*Chengqi Yan, Kai Feng, Bingkun Bao, Jing Chen, Xiang Xu, Guoyong Jiang, Yufeng Wang, Jiahe Guo, Tao Jiang, Yu Kang, Cheng Wang, Chengcheng Li, Chi Zhang, Pengjuan Nie, Shuoyuan Liu, Hans-Günther Machens, Linyong Zhu, Xiaofan Yang*, Ran Niu*, Zhenbing Chen**

1. Experimental Methods

**1.1. Isolation of milk-derived extracellular vesicles (MEVs)**

MEVs were isolated by differential centrifugation from raw milk as described.^[1, 2]^ Briefly, milk was centrifuged at 13,000 × g at 4 °C for 30 min to remove fat globules, cells and cell debris. The upper fat layer and the pellet in the bottom of the tube were discarded and the supernatant was collected. The supernatant was then centrifuged at 100,000 × g at 4 °C for 60 min to remove large particles and microvesicles. Then the supernatant was centrifuged at 140,000 × g for 90 min at 4°C and the MEV pellet was collected and washed 3 times with PBS and filtered through 0.22 μm filter membrane. MEV suspension was stored at -80 ℃ until used.

**1.2. Preparation of mangiferin-loaded MEVs (MF@MEVs)**

MEVs were loaded with mangiferin (MF, M3547, Sigma-Aldrich, Shanghai) by electroporation using the CUY21EDIT II (BEX, Japan) electroporation system. The electroporation mixture was prepared by mixing MEVs and MF in PBS, with the final concentration of MEVs in the mixture was 0.1 mg ml^-1^. The mixture was transferred into ice-cold 0.4-cm cuvettes and electroporated for 10 cycles with a perforation voltage of 110 V, a perforation opening time of 6 ms, a perforation interval of 10 ms, a penetration voltage of 25 V and a capacitance of 940 μF. Post electroporation, the mixture was transferred into a new tube at 37 ℃ for 30 min. Then, the un-loaded MF was removed and the MF@MEVs were collected through ultracentrifugation method.

The encapsulation efficiency (EE) of MF@MEVs was calculated with the following Equation (1)：

EE (%) = [(W_T_-W_S_)/W_T_] × 100% (1)

where W_T_ and W_S_ were the amounts (mg) of total MF used in the formulation and un-loaded MF remaining in the supernatant, respectively.

**1.3. Construction of glycoengineered MF@MEVs**

For glycoengineering, PNGase F and O-Glycosidase & Neuraminidase Bundle were purchased from New England BioLabs (Ipswich, MA, USA), and then we followed the manufacture’s protocol. Briefly, MF@MEV pellets were incubated with PNGase F, *O*-Glycosidase & Neuraminidase Bundle, or a combination of the two glycosidases at 37 ℃ for 24 h to remove N-glycans, O-glycans, or both of the two glycans. The resulting samples were collected by ultracentrifugation at 140,000 × g for 90 min at 4 °C and then resuspended in PBS.

**1.4. Lectin blotting analysis**

Samples of four types of MEVs in Figure 2A were resolved on 10% SDS-PAGE (Beyotime Biotechnology, Shanghai, China), transferred into the PVDF membrane (Millipore, USA). Following protein transfer, membranes were blocked for 1 h in PBS containing 5% non-fat dry milk and 0.1% Tween-20. Blots were then incubated overnight with Lectin Kit I, Biotinylated (Vector Laboratories, Burlingham, CA), and Sambucus Nigra Lectin (SNA, EBL), Biotinylated (Vector Laboratories, Burlingham, CA) at 4  °C. Then, membranes were incubated with HRP-labeled Streptavidin (Beyotime Biotechnology, Shanghai, China) for 1 h and exposed to X-ray film (UVP, USA).

**1.5. Characterization of MF@DeMEVs**

The morphology and the size distribution of MEVs in each sample were detected by transmission electron microscope (TEM, Hitachi, Japan) and NanoSight LM10 instrument (Malvern Instruments, Malvern, UK). Specific markers of EVs were detected through western blotting analysis.

**1.6. Preparation and characterization of MF@DeMEV/SA-MNPs**

Core/shell structured Fe_3_O_4_@SiO_2_ nanoparticles (S-MNPs) were purchased from Suzhou NanoMicro Technology Co. Ltd. (Suzhou, China), patch NO. MS300-SiOH. The S-MNPs were rinsed with ethanol and deionized water for 3 times to remove residual impurities before using, and 10 mg of them were sonicated in 10 mL ultrapure H_2_O for 10 min to form a homogeneous solution. Next, 10 mg 2-hydroxypropyltrimethyl ammonium chloride chitosan (HACC) with a degree of substitution of 98% (Macklin Biology Co. Ltd. Shanghai, China) was added into the S-MNP dispersion. After 24 h, the dispersion was magnetically separated, and washed with deionized water for 3 times and subsequently dispersed in 10 mL PBS solution (pH = 7.4) to obtain SA-MNPs. After that, a certain amount of SA-MNP was mixed with the MF@DeMEV solution in an oscillating incubator for 6 h to obtain MF@DeMEV/SA-MNPs.

The morphology image of MF@DeMEV/SA-MNP was recorded using a FEI Tecnai 12 transmission electron microscope at an accelerating voltage of 120 kV. Dynamic light scattering (DLS) and Zeta potentials were conducted on a BeNano 180 Zeta instrument (Bettersize Instruments Ltd.).

**1.7. In vitro release profile of MF**

The release profile of MF was detected by the dialysis method. Briefly, dialysis bags (MWCO = 3500) containing MF@DeMEV/SA-MNPs solution (3 mL) was immersed into 50 mL of PBS (pH = 7.4) at 37 °C with the shake at 100 r/min. The dialysis bags were exposed to or not to magnetic field (∇B). Sampling was performed at 1, 2, 12, 24, 36, 48, 60, 72 h time points, and the release media was replaced with fresh PBS to maintain the sink condition throughout the system. The rate of MF release was calculated using the following Equation (2):

Mangiferin Release (%) = M_R_/M_T_ × 100% (2)

where M_T_ and M_R_ were the amounts (mg) of total MF used in the formulation and released MF at each time point, respectively.

**1.8. Magnetic actuation of MF@DeMEV/SA-MNPs**

Sample cells were constructed by attaching rings of radius *R* = 10 mm and height *H* = 1 mm made from poly (methyl methacrylate) to microscopy glass slides. The sample cells were immersed in Hellmanex III solution (0.5% v/v, Hellma Analytics, Germany) for 2 h, followed by washing several times with ultrapure water. For optical observation, 320 μL MF@DeMEV/SA-MNP suspension was dropped into the sample cell, followed by covering with coverlid. The concentration of MF@DeMEV/SA-MNPs was adjusted from 0.005 to 0.1 mg mL^-1^ in order to observe their individual or collecting behavior. For magnetic actuation, magnetic fields were derived from a three-axis Helmholtz coil, which was driven by alternating current (AC and DC) signal with 0-100 Hz frequency produced by a function/arbitrary waveform generator (RIGOL DG1000Z, China) and amplified by a signal-amplifier. Videos were observed by an Axioscope 5 optical microscope (Carl Zeiss AG, Germany) and recorded by an Axiocam 208 color camera. The trajectory and instantaneous velocity of MF@DeMEV/SA-MNP in the video were analyzed by a home-written Python program. All the showing data were averaged over 10 particles.

**1.9. Numerical Simulation**

COMSOL Multiphysics software was used to simulate fluid flow around MF@DeMEV/SA-MNP when external magnetic field was applied. The Moving Mesh and Creeping Flow modules in COMSOL Multiphysics^TM^ were included in this simulation. Typically, a 200 µm × 100 µm simulation box was used. The radius of SA-MNP and MF@DeMEV were 250 nm and 75 nm, respectively. The fluid water had a mass density of 1.0 ×10^3^ kg m^-3^, viscosity of 8.9 × 10^-4^ Pa s, and relative permittivity of 78.4 (*T* = 298.15 K). For magnetic actuation, the MF@DeMEV/SA-MNP moved in a constant direction and speed. In this simulation, the velocity of MF@DeMEV/SA-MNP was set to 2 μm s^-1^ as a Dirichlet boundary condition.

**1.10. 3D collagen gel penetration assay**

Collagen gels were prepared as previously reported.*[3]* Briefly, 0.2 ml collagen stock solution (5 mg ml^-1^ collagen type I, rat tail, solarbio) was added to 2 or 1 ml sterile ddH_2_O with PBS (10×) (Gibco) and 1 M NaOH were added to adjust pH. Final gel concentration was 0.5 or 1 mg ml^-1^. Collagen solutions were pipetted on one side of a sample cell of radius R = 10 mm and height H = 1 mm so that they only took up one-third of the total cell volume. Gels were placed in a temperature-controlled incubator at 37 °C and 5% CO_2_ for 30 min for polymerization. After polymerization, gels were thoroughly washed with fresh PBS (pH 7.4) several times to remove unreacted species and set the pH to neutral, and the rest of the chamber was filled with PBS solution. Next, Dil-labeled MF@DeMEV/SA-MNP were pipetted at the far end of the chambered coverslip. The setup was incubated 30 min with the permanent magnet setup aligning the MF@DeMEV/SA-MNP toward the collagen gels on one corner of the chamber. Quantification of invaded MF@DeMEV/SA-MNP within the collagen gels was characterized by optical microscope and confocal microscope (Olympus, FV1200, Japan). A minimum of six different images were used for the quantification of the collagen gel invasion.

**1.11. Cell culture**

Human umbilical vein endothelial cells (HUVECs) (#GDC166, CCTCC) were obtained from the China Center for Type Culture Collection (CCTCC, Wuhan, China) and grown in high-glucose Dulbecco’s modified Eagle’s medium (DMEM; Gibco, USA) supplemented with 10% fetal bovine serum (FBS; Gibco, USA). Primary mouse dermal fibroblasts were isolated from the skin of C57BL/6J mice using previously described protocols and cultured in DMEM with 10% FBS.*[4]* All cells were incubated at 37°C under humidified atmosphere containing 5% CO_2_.

**1.12. *In vitro* cell viability assay**

To test *In vitro* cytotoxicity of MF@DeMEV/SA-MNPs, endothelial cells and fibroblasts were seeded into 96-well plates for 24 h. Then, they were co-cultured with different concentrations of MF@DeMEV/SA-MNP (50, 100, 150, 200 and 250 µg mL^-1^). After incubation for another 48 h and 72 h, the cytotoxicity was measured using the CCK-8 solution according to the manufacturer’s instructions. The absorbance of the solution at 450 nm was recorded using a microplate reader (TECAN, Switzerland).

To test the effect of MGO on cell viability, endothelial cells and fibroblasts were seeded into 96-well plates for 24 h. Then, they were co-cultured with different concentrations of MGO (0, 200, 400, 600, 800 and 1000 µM). After incubation for another 48 h, the cell viability was measured using the CCK-8 solution according to the manufacturer’s instructions. The absorbance of the solution at 450 nm was recorded using a microplate reader (TECAN, Switzerland).

**1.13. Hemocompatibility assay**

To evaluate the hemocompatibility of MF@DeMEV/SA-MNPs with red blood cells (RBCs), RBCs were collected from blood after centrifugation at 2000 rpm for 10 min. Purified RBCs were washed thrice and diluted to a concentration of 5% (v/v). The diluted RBC suspensions (200 µL) were mixed with MF@DeMEV/SA-MNPs to obtain 700 µL of MF@DeMEV/SA-MNP mixtures of different concentrations (50, 100, 150, and 200 µg mL^-1^). Ultrapure H_2_O was used as the positive control, and PBS was used as the negative control. After incubation at 37°C for 4 h, the mixtures were centrifuged at 2000 rpm for 10 min. The supernatant (100 µL) was transferred to a 96-well microplate, and the absorbance at 540 nm was recorded using a microplate reader (TECAN, Switzerland). Hemolytic quantification was performed with the following Equation: (3)

Hemolysis (%) = [(As– An)/(Ap–An)] × 100% (3)

where As, Ap, and An were the absorbance of the supernatant treated with MF@DeMEV/SA-MNPs, the positive control, and the negative control, respectively

**1.14. *In vitro* cellular uptake assay**

For fluorescence labeling, untreated or glycosidase treated MF@MEVs were incubated with an red fluorescent dye, Dil (Biotium, USA), for 30 min and then centrifuged to remove contaminating dye. Next, these Dil-labeled EVs were synthesized with SA-MNPs to obtain different types of Dil-labeled nanorobots.

For flow cytometry, endothelial cells and fibroblasts were seeded in 6-well culture plates for 12 h, after treated by PBS or MGO for another 24 h, they were co-cultured with different types of Dil-labeled nanorobots with the same quality for 24 h. Then, the cells were washed twice with PBS and harvested. The cellular uptake of these nanorobots was determined by measuring the mean fluorescence intensity (MFI) using Gallios Flow Cytometer (Beckman Coulter, Miami, FL, USA) according to the manufacturer’s instructions. Data were analyzed using the FlowJo software (version 10.8.1, BD biosciences).

For confocal microscopy analysis, endothelial cells and fibroblasts were seeded in 24-well culture plates for 12 h, after treated by PBS or MGO for another 24 h, they were co-cultured with Dil-labeled MF@MEV/SA-MNPs or MF@DeMEV/SA-MNPs with the same quality for 24 h. After incubation, cells were washed twice with PBS and fixed in 4% paraformaldehyde for 10 min; thereafter, the nucleic was stained with DAPI (Solarbio, Beijing, China) and the cytoskeleton was stained with FITC phalloidin (Yeasen Biotech Co., Shanghai, China) according to the manufacturer’s instructions. The cellular uptake was observed by using the confocal laser scanning microscope (CLSM).

**1.15. Cellular proliferation, migration and tube formation assay**

Endothelial cells and fibroblasts from different treated groups were grown in 96-well culture plates for 12 h. After 2 h incubation with the EdU, the proliferation rates of the cells from each group were evaluated with Cell-Light EdU Apollo In Vitro Kit (Ribobio, Guangzhou, China).

For in vitro wound healing assay, endothelial cells and fibroblasts were seeded in 24-well plates for 24 h to form a confluent monolayer. Then the cell sheet was wounded through scratching the culture well surface with a 200 μL pipette tip. The ‘‘scratch wound’’ creates a cell-free denuded space that the remaining cells can migrate over. The migration ability of cells from different treated groups was monitored by microscopy.

Endothelial cells from different treated groups were seeded in 96-well culture plates (25,000 cells per well) that had been coated with 70 μL Matrigel Basement Membrane Matrix (BD Biosciences, CA, USA). Tube formation was detected under microscopy at 6 h incubation. Total tube length of the endothelial tubes was measured by angiogenesis plugin available from ImageJ.

**1.16. ROS generation, lipid peroxidation, and antioxidant enzyme activity assay**

For intracellular ROS generation assay, endothelial cells and fibroblasts were seeded in 6-well culture plates and treated with different treatments for 24 h. Intracellular ROS levels were detected using a ROS Assay Kit (Beyotime Biotechnology, Shanghai, China) according to the manufacturer’s instructions. Briefly, the cells were stained with DCFH-DA dye for 30 min, and then washed twice with PBS and analyzed by Gallios Flow Cytometer (Beckman Coulter, Miami, FL, USA). Data were analyzed using the FlowJo software (version 10.8.1, BD biosciences).

For lipid peroxidation assay, supernatants from different treated endothelial cells and fibroblasts were collected. The levels of malondialdehyde (MDA) in the supernatants were measured using a lipid peroxidation MDA assay kit (Beyotime Biotechnology, Shanghai, China) following the manufacturer's instructions. In brief, 200 μL MDA solution was added to each supernatant or standard sample, the mixture was heating at 100°C for 15 min and then centrifuged at 1,000 g for 10 minutes to harvest the supernatant. The MDA levels were detected using UV-Vis spectrophotometry.

The activity of SOD and GPx were tested using commercially available kits (Beyotime Biotechnology, Shanghai, China) following the manufacturer's instructions. Briefly, endothelial cells and fibroblasts were homogenized in ice-cold 0.1  M phosphate buffer (pH 7.4), then the homogenates were filtered and centrifuged using a refrigerated centrifuge at 12,000 × g for 20  min at 4  °C. The obtained supernatants were used to determine the SOD and GPx enzyme activity.

**1.17. Western blotting analysis**

Isolation of nuclear protein was performed according to Nuclear and Cytoplasmic Protein Extraction Kit (Beyotime Biotechnology, Shanghai, China). Total protein was extracted by RIPA lysis buffer with proteinase inhibitor (Roche, Switzerland). Equal amount of protein (20-40 μg) was separated by SDS-PAGE (Beyotime Biotechnology, Shanghai, China), transferred into the PVDF membrane (Millipore, USA), and then incubated overnight with primary antibodies specific for Nrf2 (#16396-1-AP, Abcame), Lamin B1 (#66095-1-lg, Proteintech), NQO1 (#11451-1-AP, Proteintech), HO-1 (#10701-1-AP, Proteintech), Beta Actin (#66009-1-lg, Proteintech). Then, the membrane was incubated with secondary antibodies (Aspen, China) for 1 h and exposed to X-ray film (UVP, USA).

**1.18. *In vitro* antibacterial evaluation**

The gram-positive bacteria Staphylococcus aureus (*S. aureus*) and the gram-negative bacteria Escherichia coli (*E. coli*) were obtained from the School of Life Sciences, Hubei University. The bacteria were suspended in standard Luria-Bertani (LB) medium without antibiotics and placed in a shaking incubator for 220 rpm at 37 °C for 16 h. The absorbance of the bacterial solution at 600 nm was recorded using a microplate reader (TECAN, Switzerland).

For the plate count method, PBS, S-MNPs, SA-MNPs, or MF@DeMEV/SA-MNPs were added into *S. aureus* (10^6^ CFU mL^-1^) and *E. coli* (10^6^ CFU mL^-1^). Subsequently, these bacterial suspensions were incubated at 37 °C for 12 h. Then the suspensions were diluted with PBS to 10^3^ CFU mL^-1^, and 100 µL of the dilution was plated on LB agar. After 12 h of incubation, the number of CFUs was counted.

For the live/dead bacterial staining assay, PI dye and 4′,6diamidino-2-phenylindole (DAPI) were used to detect dead or total bacteria, respectively. The stained bacteria were dropped on clear glass slides and imaged using a fluorescence microscope (IX71, Olympus).

For the in vitro anti-biofilm efficacy, *S. aureus* suspensions (10^8^ CFU mL^-1^) or *E. coli* suspensions (10^8^ CFU mL^-1^) were added in 96-well tissue culture plates and incubated at 37 °C after 48 h to form biofilms. Followed by washing with PBS to remove free bacteria, PBS, S-MNPs, SA-MNPs, or MF@DeMEV/SA-MNPs were added into *S. aureus* and *E. coli* biofilms with or without magnetic field (∇B) for 12 h. Then, biofilms were washed with PBS and stained with crystal violet solution (0.1 %) for 30 min. The stained biofilms were photographed and incubated with 200 μL of ethanol (95 %). The absorbances at 590 nm were detected by a microplate reader (TECAN, Switzerland) to calculate the biofilm residual.

**1.19. Infected diabetic wound model**

All animal experiments were approved by the Animal Care Committee of Tongji Medical College. After a 12 h fast, eight week-old male C57BL/6 mice were intraperitoneally injected with streptozotocin (STZ, 50 mg kg^-1^) for 5 days, and after 2 weeks, the blood glucose was measured by a blood glucose monitor. Diabetic mice were successfully induced when the blood glucose was above 16.7 mM and maintained for another 4 weeks before full-thickness cutaneous wounds were formed. Before surgery, diabetic mice were anesthetized with pentobarbital sodium (Sigma-Aldrich) (1%, 50 mg kg^-1^). After shaving and sterilization, full-thickness excision wound at diameter of 8 mm was performed on the back of all mice. Then, 50 µL of *S. aureus* suspension (2×10^7^ CFU mL^-1^) was added on each wound area and retained for 2 days. Subsequently, the diabetic mice with successful infected wound model were randomly divided into 7 groups (n = 10): control (PBS) group, free MF group, SA-MNP+∇B group, DeMEV/SA-MNP+∇B group, MF@MEV/SA-MNP+∇B group, MF@DeMEV/SA-MNP group, and MF@DeMEV/SA-MNP+∇B group. Treatments were performed after the infected wound models were created and on day 0, 4, and 8. Digital photographs were taken on day 0, 4, 8, and 12, and the wound areas were measured using the Image J software.

**1.20. Histological and immunofluorescence analysis**

On day 12 post-treatment, the whole wound bed of each mice was obtained for histological analysis. The wounds were fixed with 4 % paraformaldehyde. After being dehydrated with a series of graded ethanol, the tissues were then embedded in paraffin and cut into 8 μm thick longitudinal sections before further staining. The length of un-epithelialization and the width of wound bed were analysed by using hematoxylin and eosin (H&E) staining. The degree of collagen accumulation was evaluated by Masson staining.

To determine angiogenesis of wound beds, the sections were incubated with CD31 (#YZU0122121, R&D), and α-SMA antibody (#14395-1-AP, Proteintech) overnight at 4 °C. After being washed three times with PBS, the sections were incubated with a second antibody (Aspen, China) for 1 h at room temperature. The image was taken by a microscope, and then analyzed by using ImageJ software.

**1.21. Detection of wound ROS levels**

After the wound tissue samples were harvested, they were processed into cryosections and stained with DHE. Images were obtained using a fluorescence microscope and analyzed using ImageJ software.

**1.22. *In vivo* biocompatibility assay**

Primary organs (heart, liver, spleen, lung, and kidney) were obtained from the mice. After fixing with 4 % paraformaldehyde, the organs were embedded in paraffin and cut into 5-μm-thick sections. The sections were stained with hematoxylin and eosin (H&E) and imaged using a light microscope (IX71, Olympus).

**1.23. Statistics**

All statistical analyses were performed using GraphPad Prism software (version 8.0, La Jolla, CA, USA). For the comparison of two groups, unpaired Student’s t test was applied. One-way analysis of variance (ANOVA) were carried out for multiple group comparisons. All data were presented as mean ± (SD). Statistical significance was set at p < 0.05.

**2. Supplementary Figures**


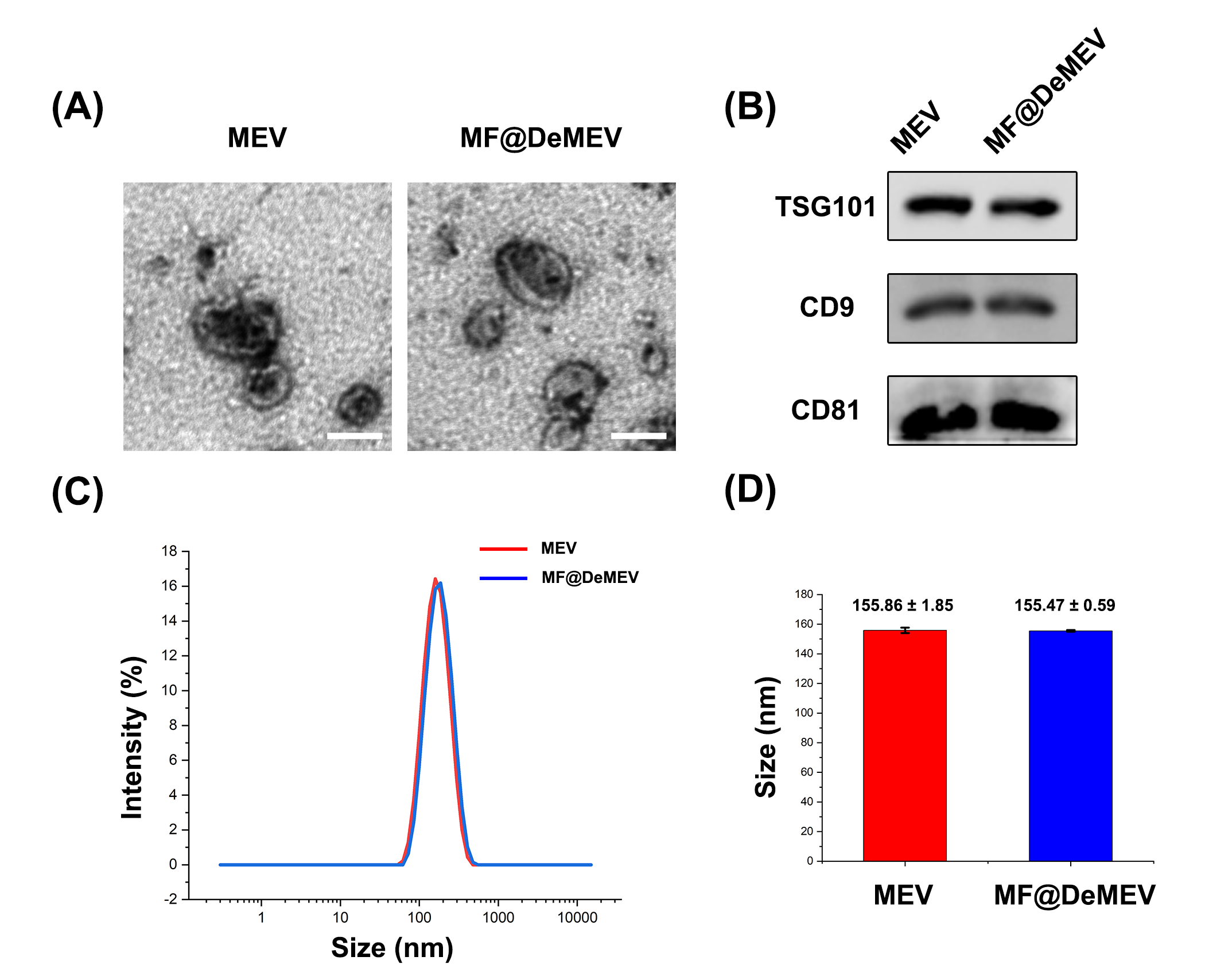


**Figure S1** Characterization of MF@DeMEVs. (A) TEM identified the morphology of MEVs and MF@DeMEVs. Scale bar: 100 nm. (B) Western blotting analysis of EV specific markers including TSG101, CD9 and CD81 of MEVs and MF@DeMEVs. (C, D) NTA identified the size distribution of MEVs and MF@DeMEVs. Data were presented as Mean ± SD.


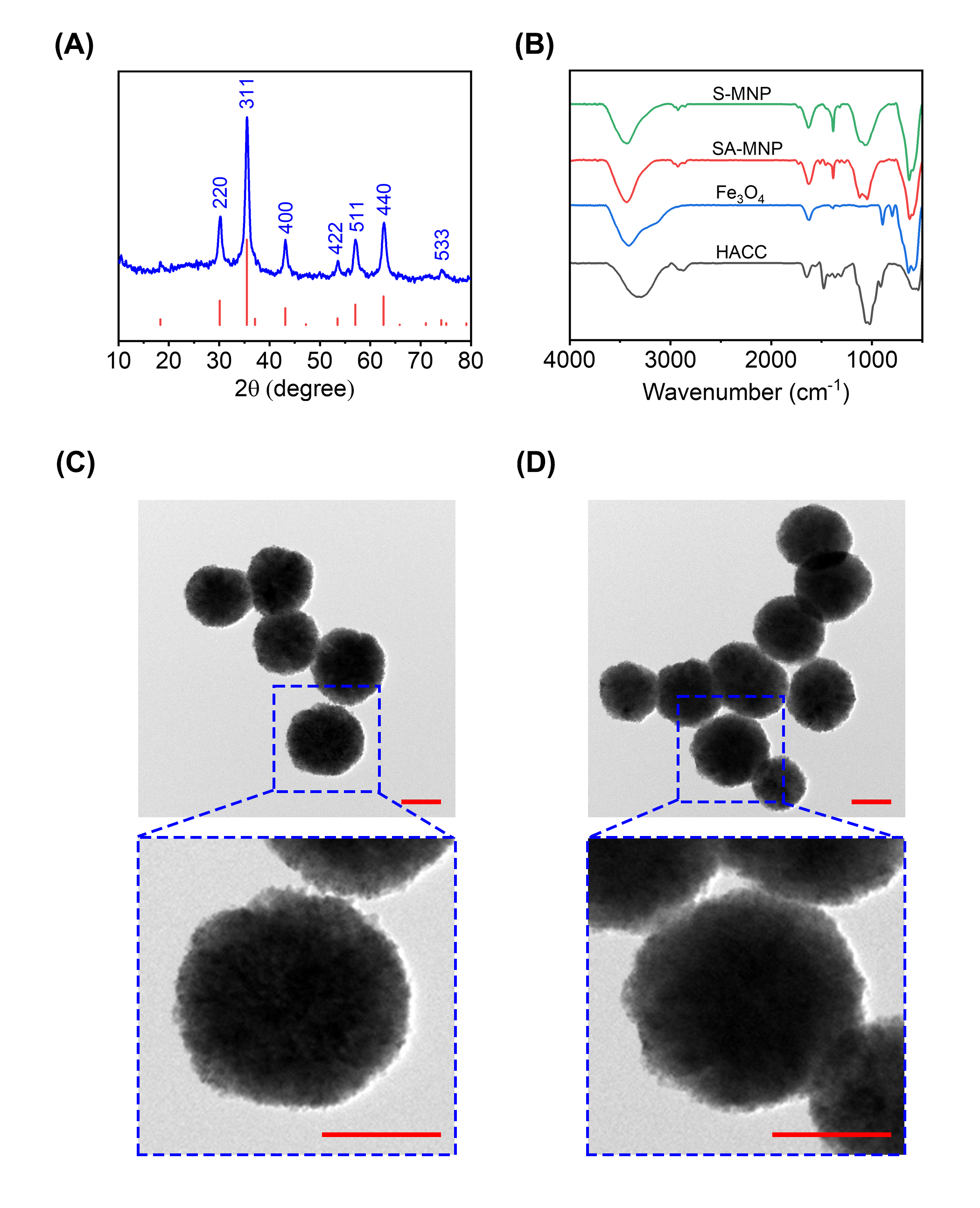


**Figure S2** Characterization of S-MNPs and SA-MNPs. (A) XRD spectrum of S-MNP. (B) FTIR spectra of S-MNP, SA-MNP and control samples. (C, D) TEM identified the morphology of S-MNPs (C) and SA-MNPs (D). Scale bar: 200 nm.


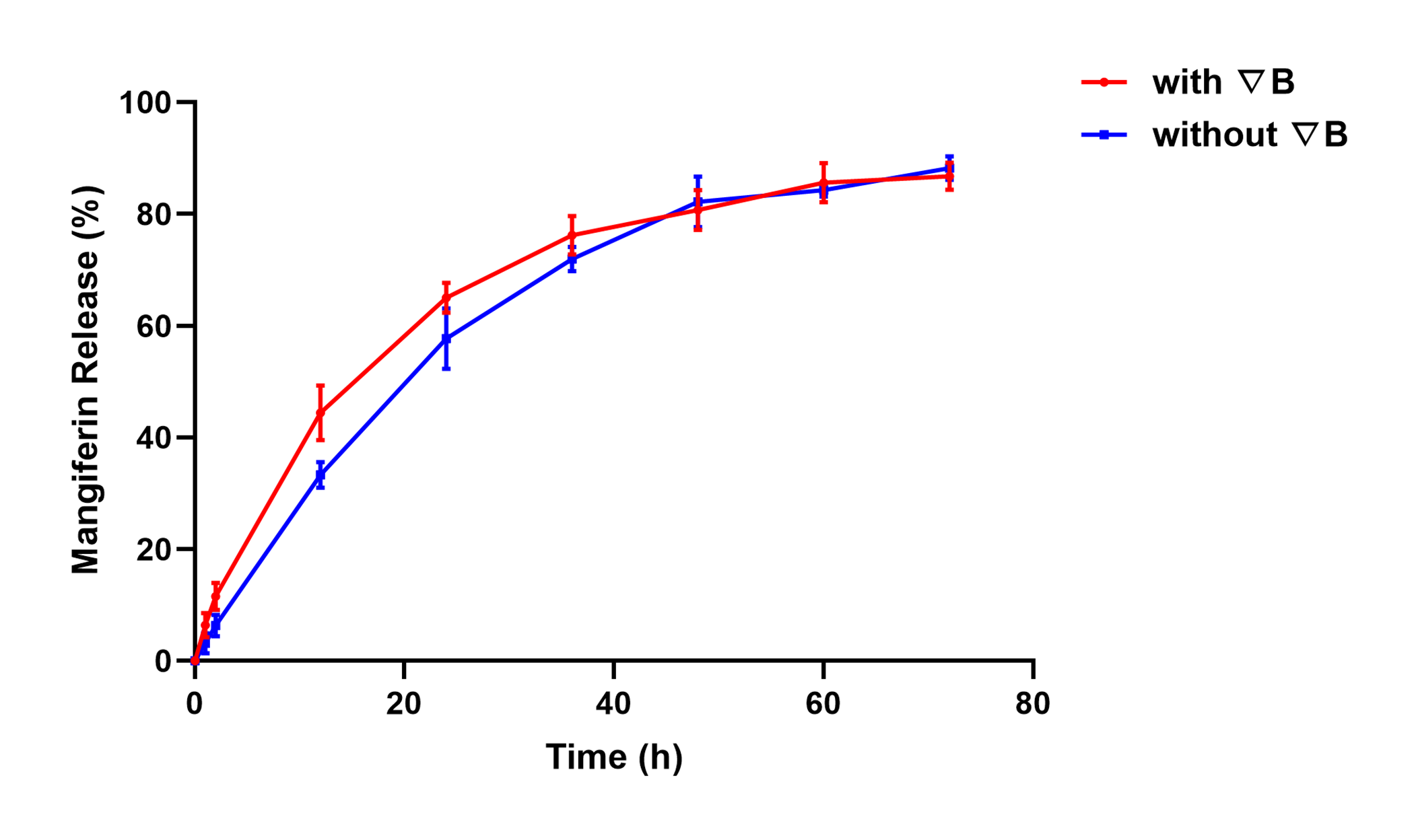


**Figure S3** Drug release behavior of MF@DeMEV/SA-MNPs with or without external magnetic fields (∇B).


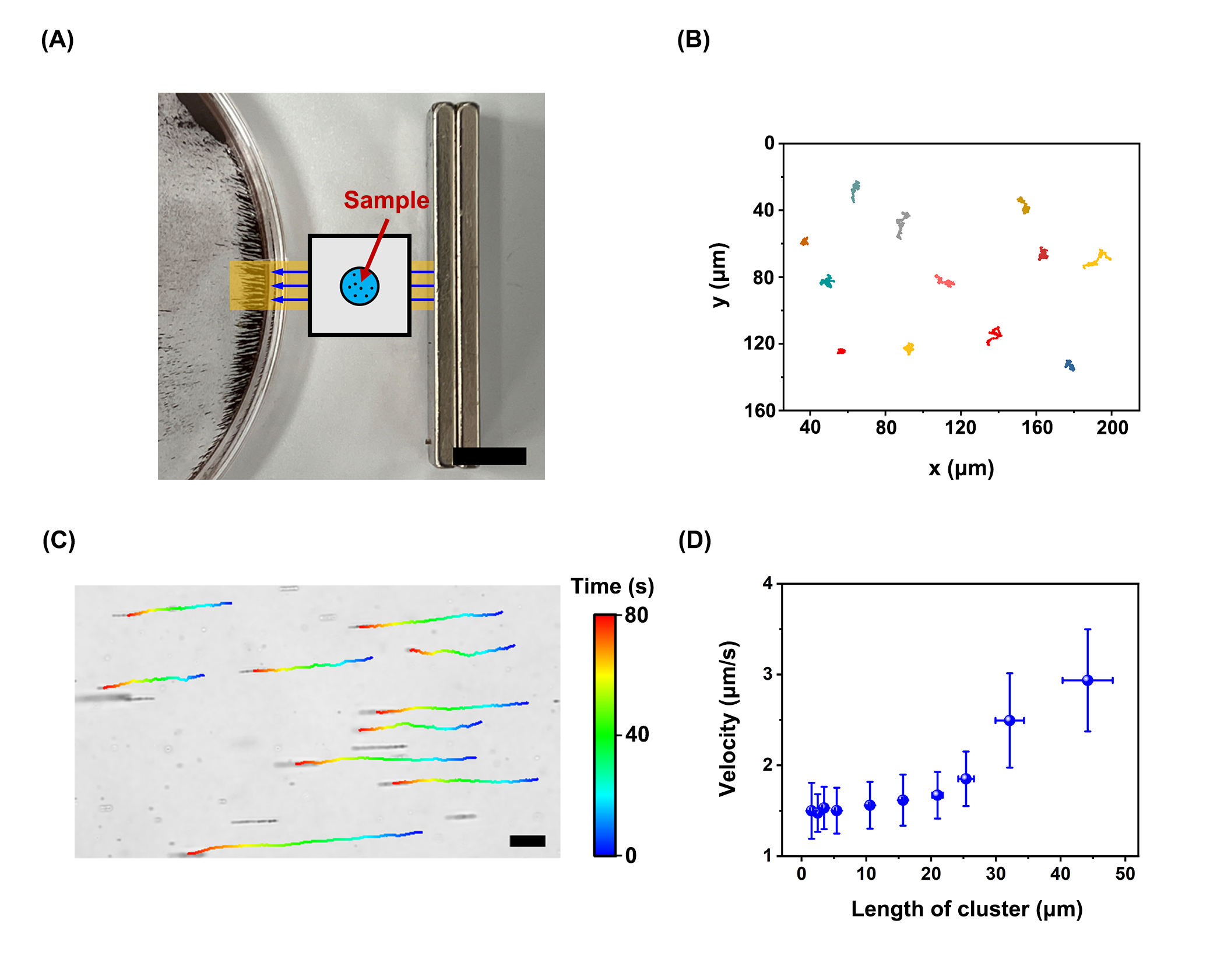


**Figure S4** Magnetic actuation of MF@DeMEV/SA-MNPs. (A) Magnetic induction line of the used magnet. Scale bar: 1  cm. (B) Trajectories of individual MF@DeMEV/SA-MNP in Brownian motion. (C, D) Trajectories within 80 s (C) and mean velocity (D) of MF@DeMEV/SA-MNP chain-like swarms of different lengths controlled by a gradient magnetic field of strength 10 mT (measured at the nanorobot’s site). Scale bar: 20  μm.


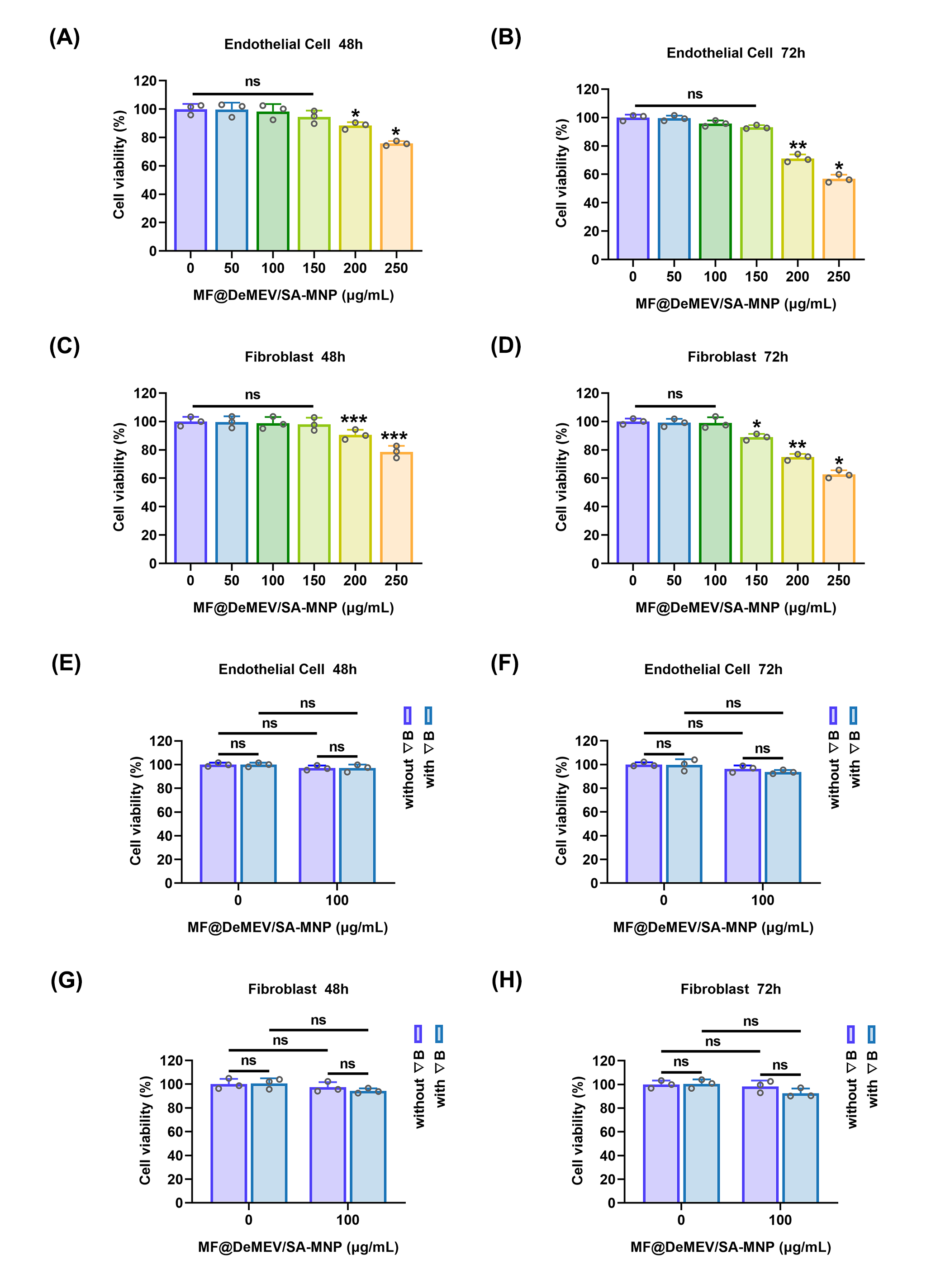


**Figure S5** The cytotoxicity of MF@DeMEV/SA-MNPs *in vitro*. Cell viability of endothelial cells (A, B) and fibroblasts (C, D) after treatment with different concentrations of MF@DeMEV/SA-MNPs for 48 h or 72 h measured by CCK-8 assay (n = 3). Cell viability of endothelial cells (E, F) and fibroblasts (G, H) after treatment with 0 or 100 μg mL^-1^ of MF@DeMEV/SA-MNPs exposed to or not to magnetic fields (∇*B*) for 48 h or 72 h measured by CCK-8 assay (n = 3). Data were presented as Mean ± SD; ns no significant, * p < 0.05, ** p < 0.01, *** p < 0.001.


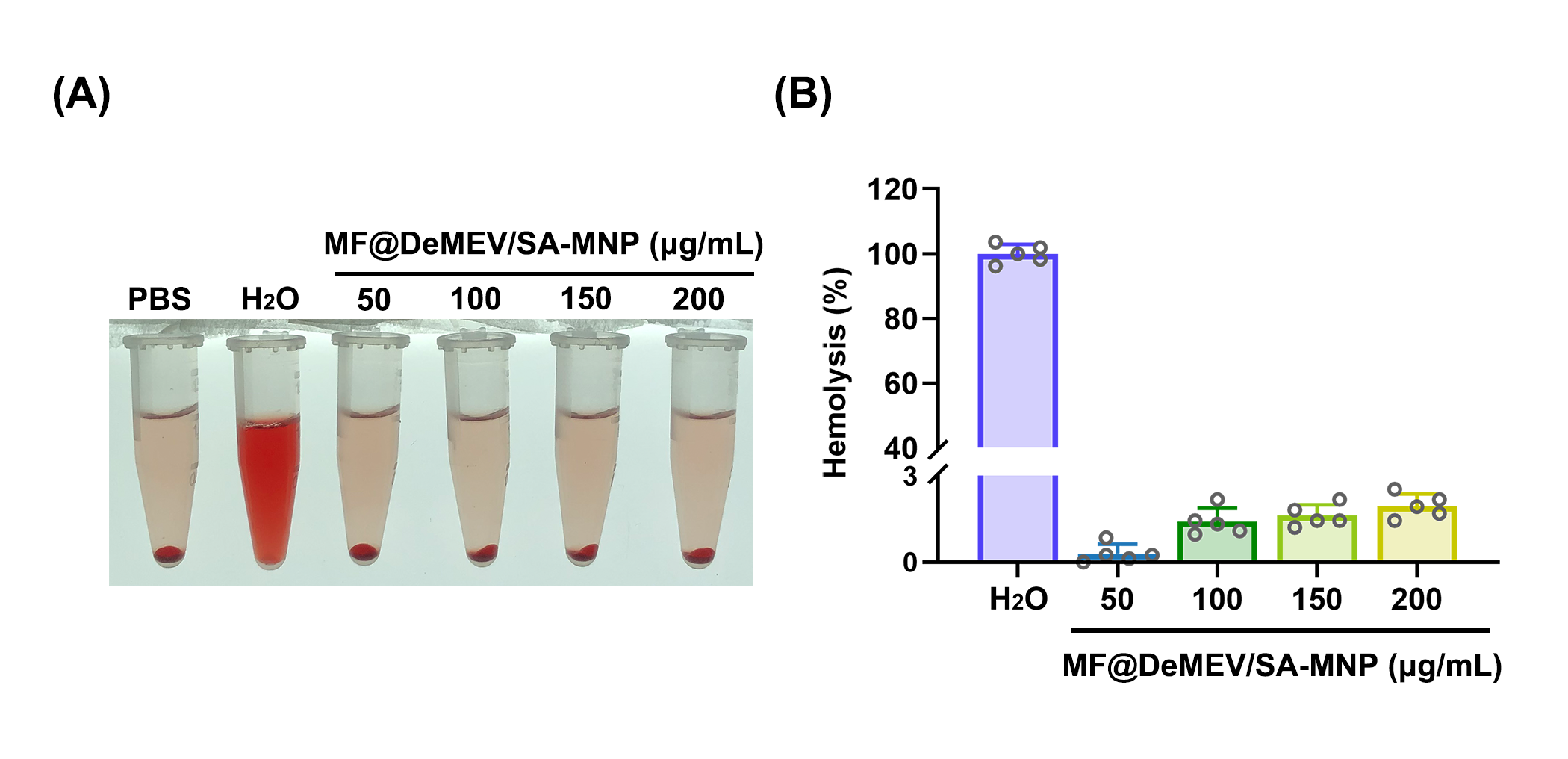


**Figure S6** The hemolysis test of MF@DeMEV/SA-MNPs in vitro. The image (A) and ratios (B) of hemolysis for MF@DeMEV/SA-MNPs with different concentrations (n = 3).


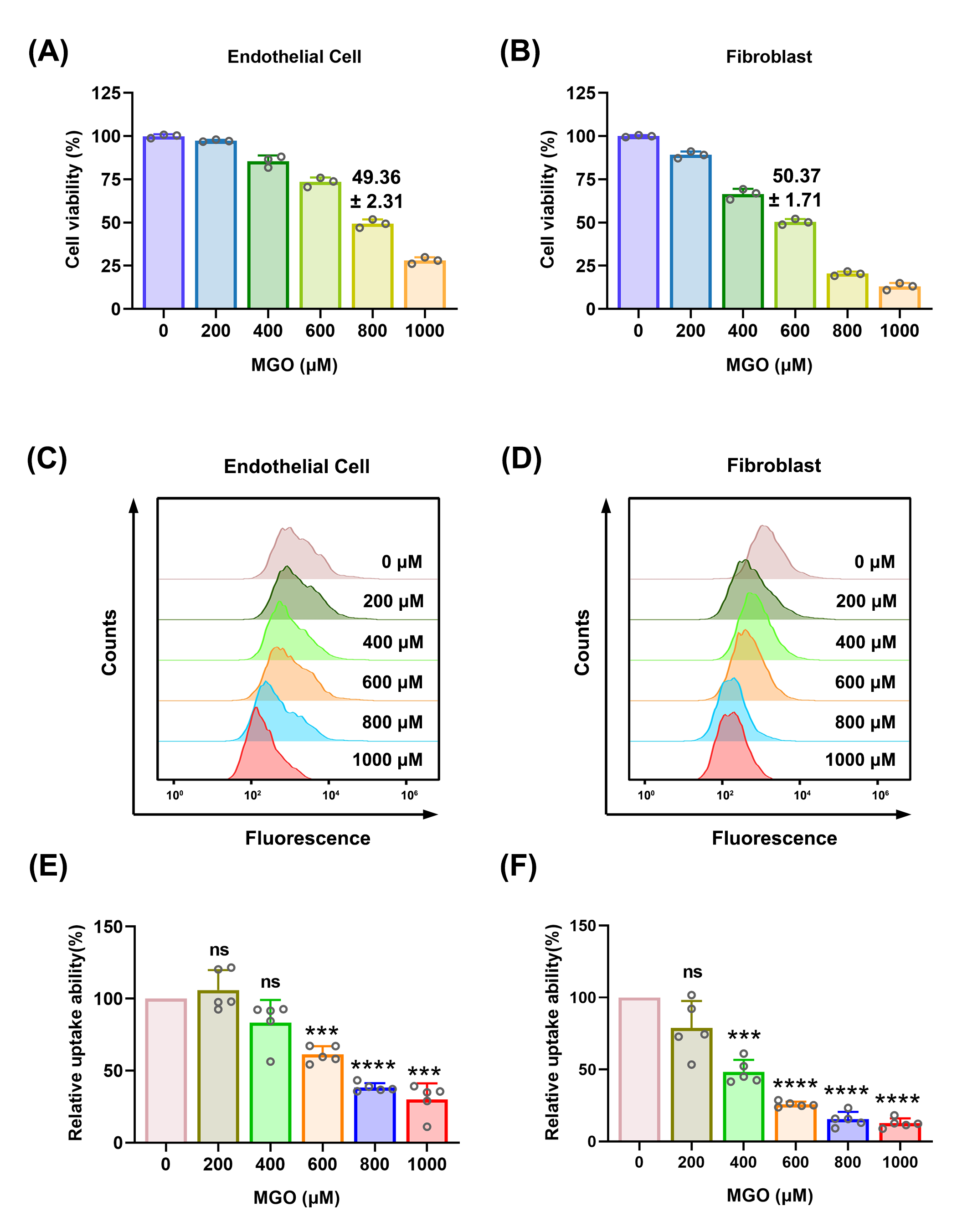


**Figure S7** Damage to cell functions caused by MGO. (A, B) Cell viability of endothelial cells (A) and fibroblasts (B) after treatment with different concentrations of MGO measured by CCK-8 assay (n = 3). (C-F) Cellular uptake efficiency of MF@MEVs in endothelial cells (C, E) and fibroblasts (D, F) after treatment with different concentrations of MGO detected by flow cytometry (n = 5; ns no significant, *** p < 0.001, **** p < 0.0001 vs. MGO = 0 µM). Data were presented as Mean ± SD.


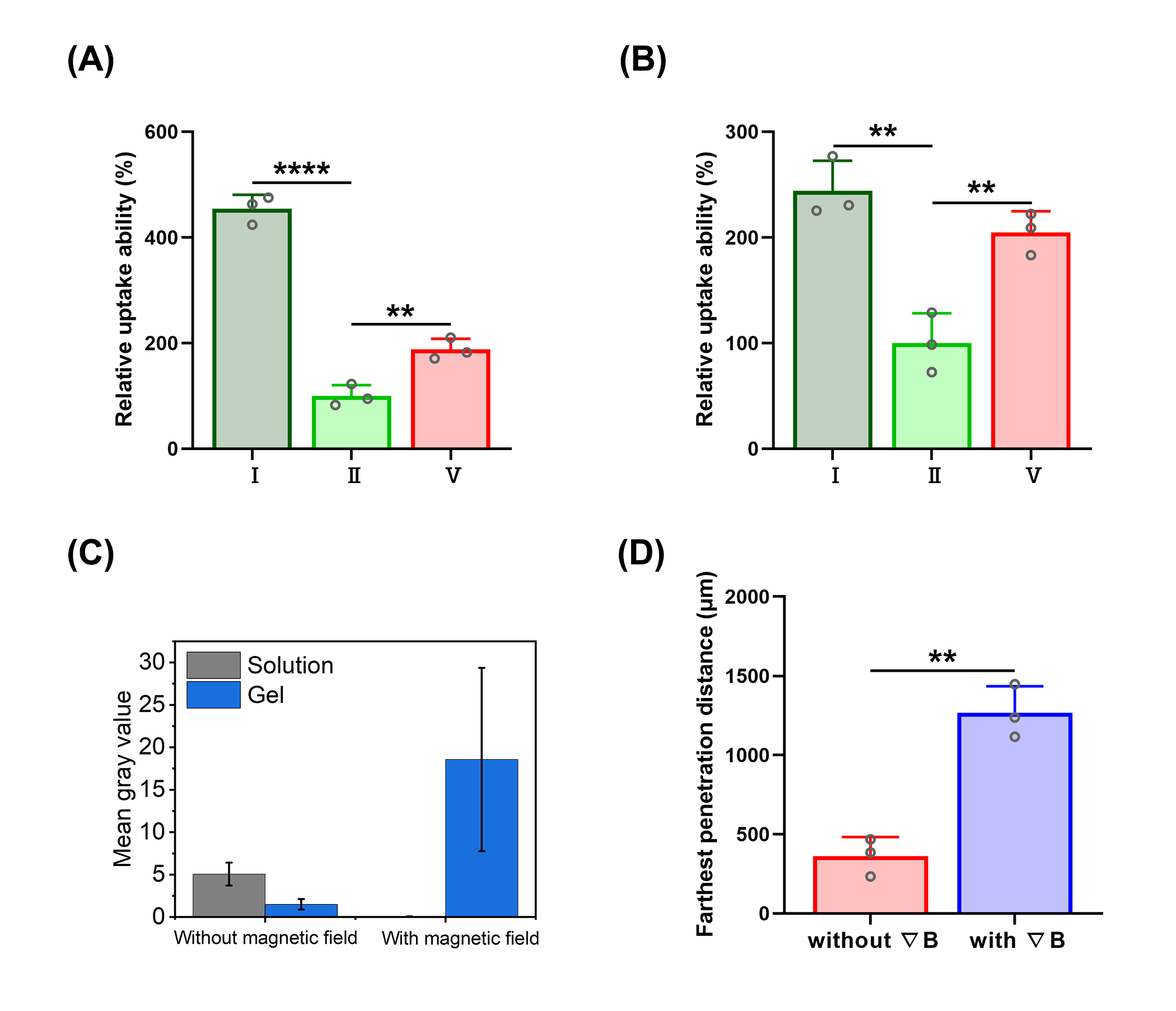


**Figure S8** Quantification analysis of the CLSM images in Figure 3E (A), Figure 3F (B), and Figure 3J (C). Quantification analysis of the farthest penetration distance of MF@DeMEV/SA-MNPs in the dermis layer of wound tissue with or without external magnetic fields (∇*B*) (D).


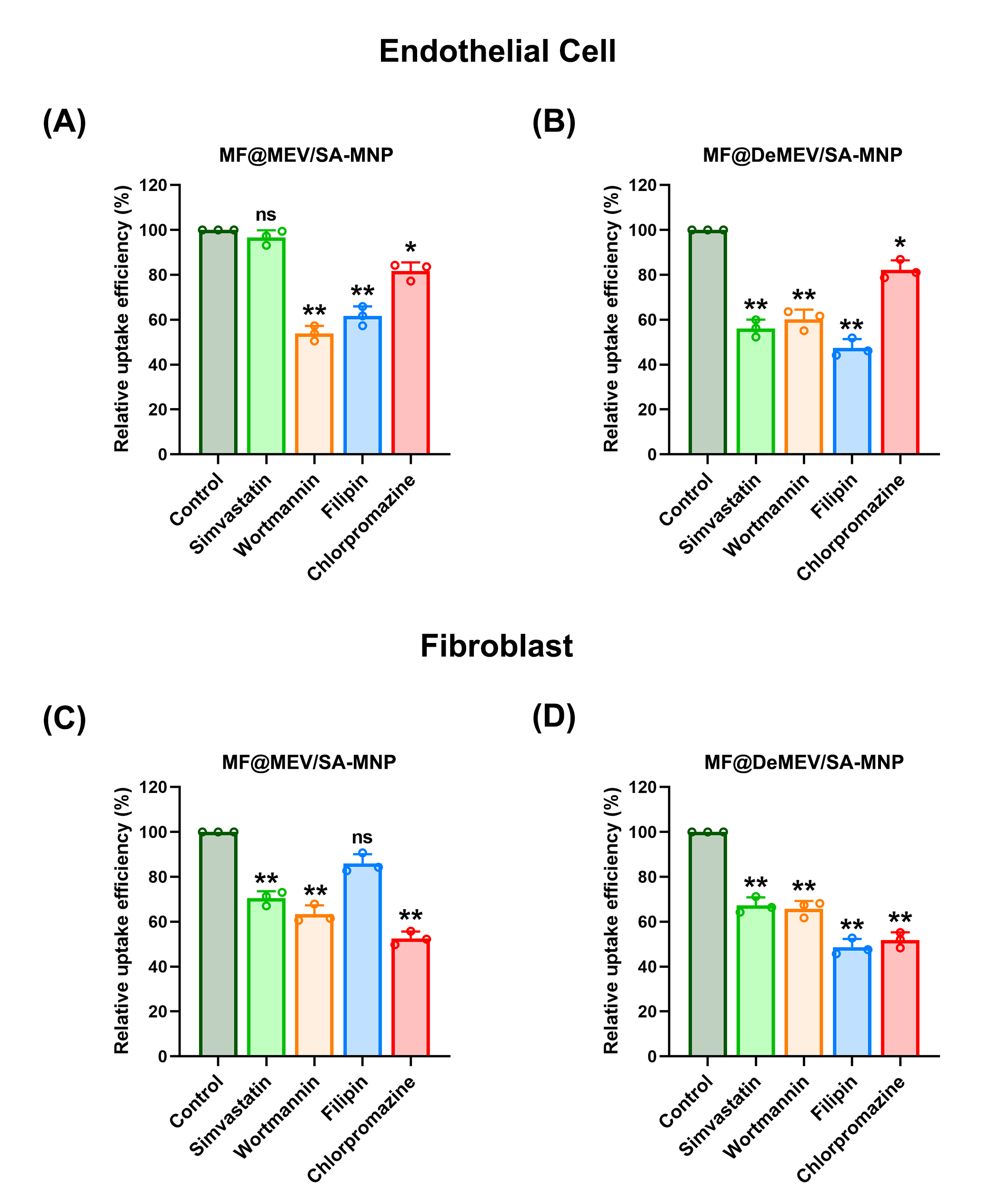


**Figure S9** Cellular uptake efficiency of MF@MEV/SA-MNPs and MF@DeMEV/SA-MNPs in endothelial cells (A, B) and fibroblasts (C, D) pretreated with different internalization inhibitors detected by flow cytometry. Cells without pretreatment with any inhibitors were taken as a control (n = 3; ns no significant, * p < 0.05, ** p < 0.01 vs. Control). Data were presented as Mean ± SD.


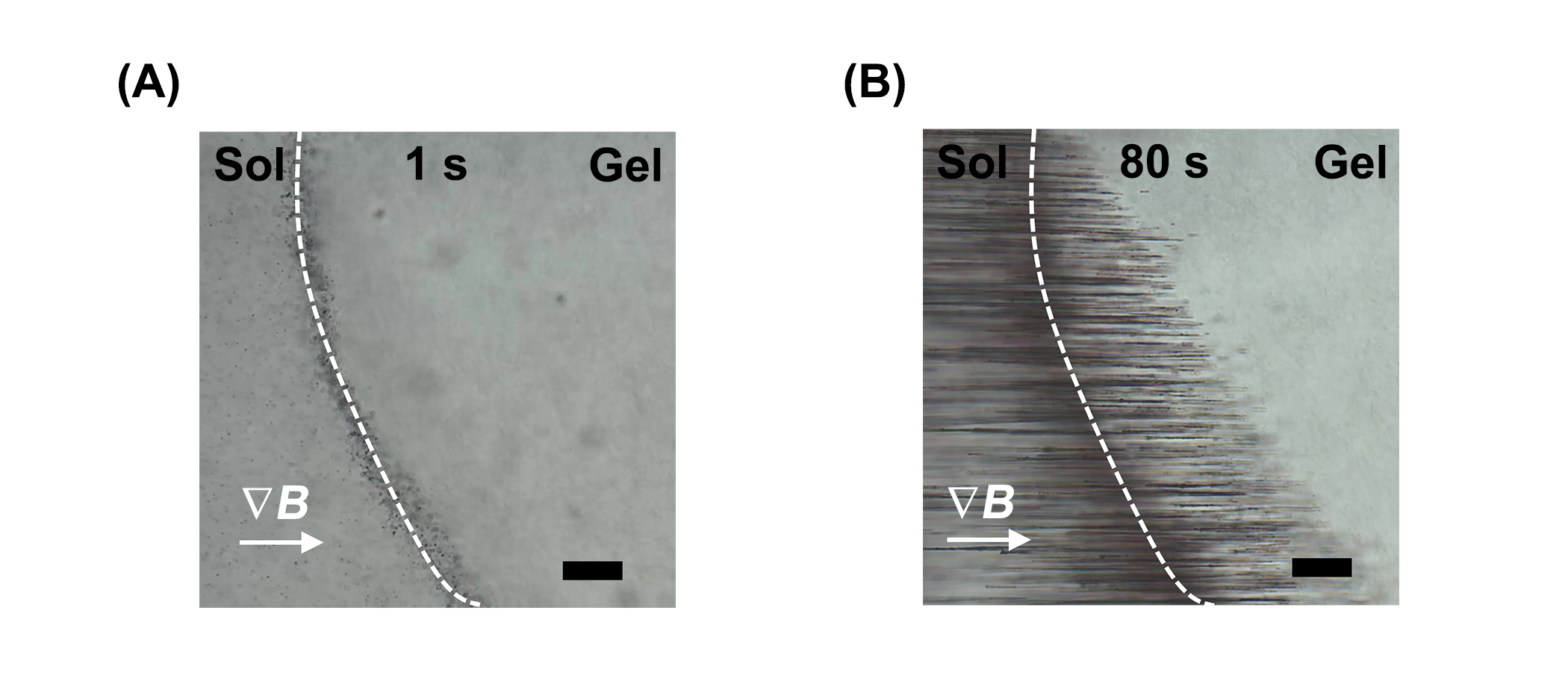


**Figure S10** Digital photographs of MF@DeMEV/SA-MNPs penetrating into the gel at (A) 1 s and (B) 80 s. Scale bar: 50  μm.


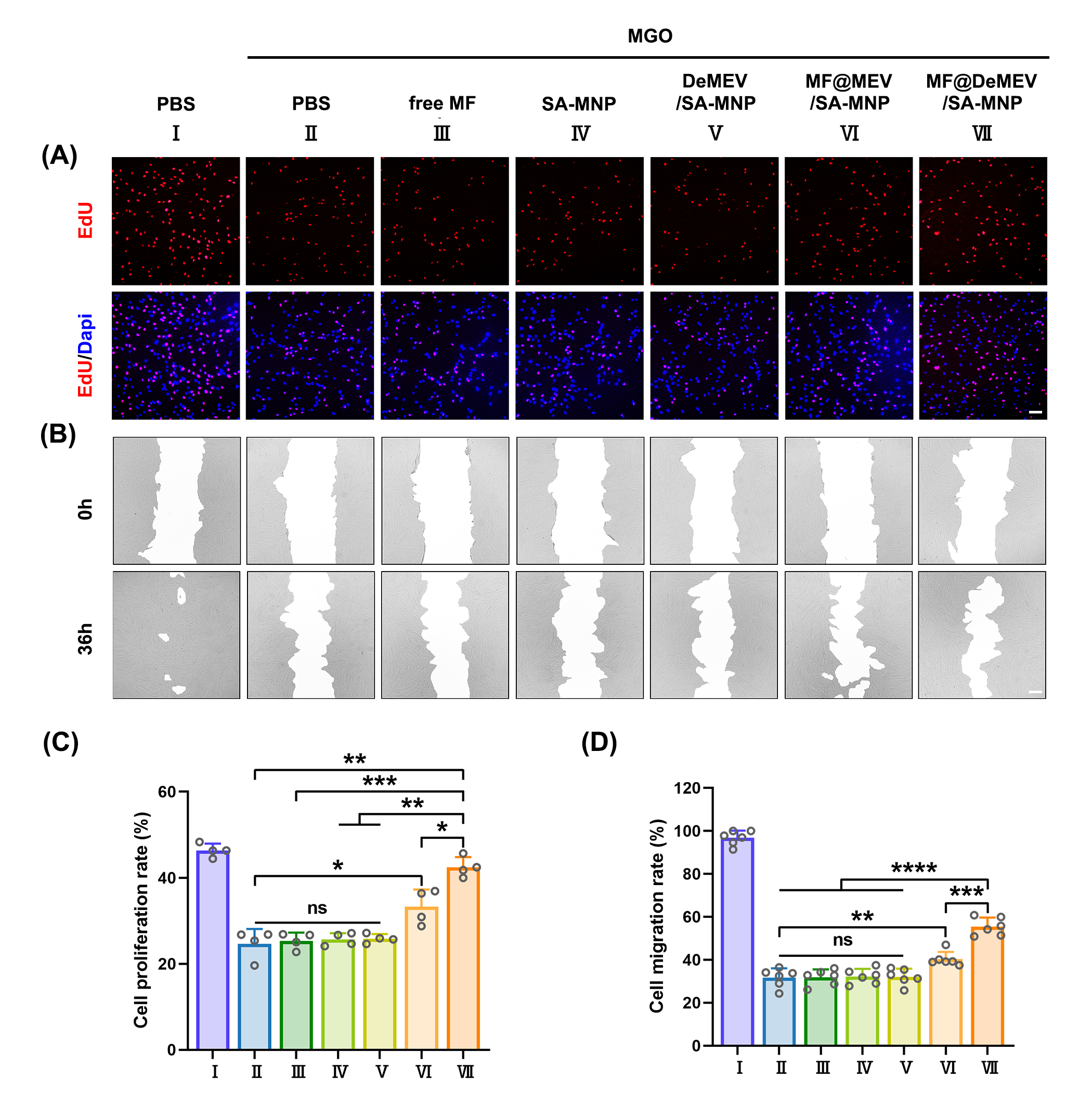


**Figure S11** MF@DeMEV/SA-MNPs reversed fibroblast function impaired by oxidative damage. (A, C) The proliferation rate of fibroblasts after different treatments analyzed by EdU assay. Red and blue fluorescence represented proliferative cells and cellular nuclei, respectively (n = 4). Scale bar: 50 μm. (B, D) The migration rate of fibroblasts after different treatments analyzed by wound healing assay (n = 6). Scale bar: 100  μm. Data were presented as Mean ± SD; ns no significant, * p < 0.05, ** p < 0.01, *** p < 0.001, **** p < 0.0001.


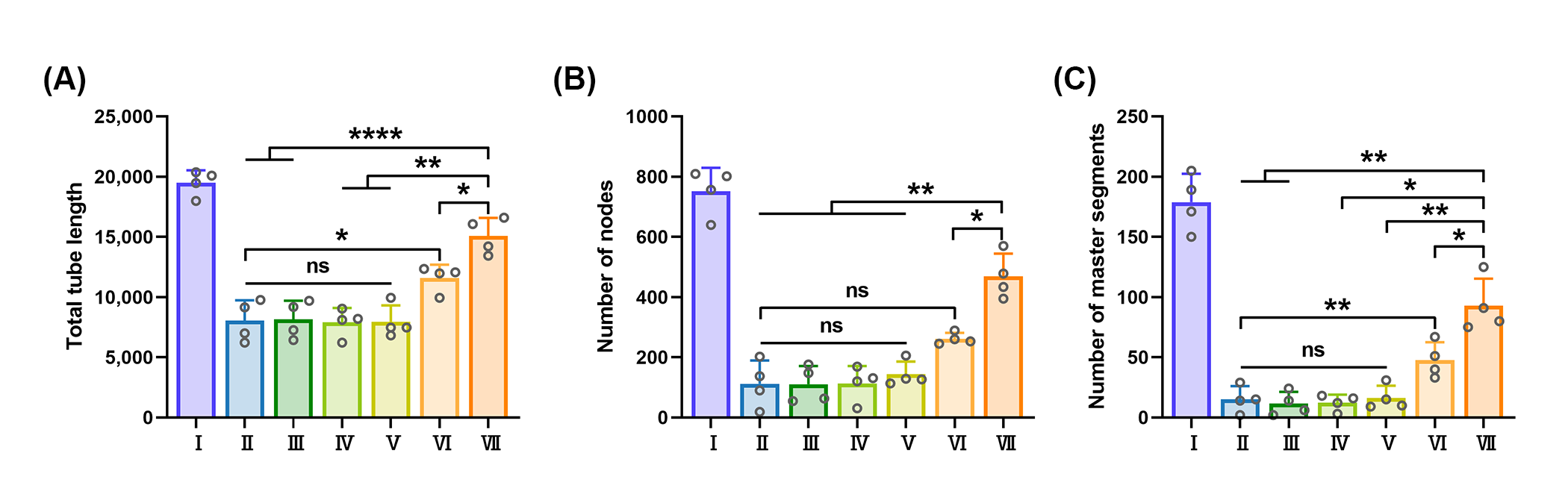


**Figure S12** Quantification analysis of vascular network formation assay in Figure 4C. Statistical analysis of tube formation ability of endothelial cells in Figure 4C using total tube length (A), number of nodes (B), and number of master segments (C) as indicators (n = 4). Data were presented as Mean ± SD; ns no significant, * p < 0.05, ** p < 0.01, **** p < 0.0001.


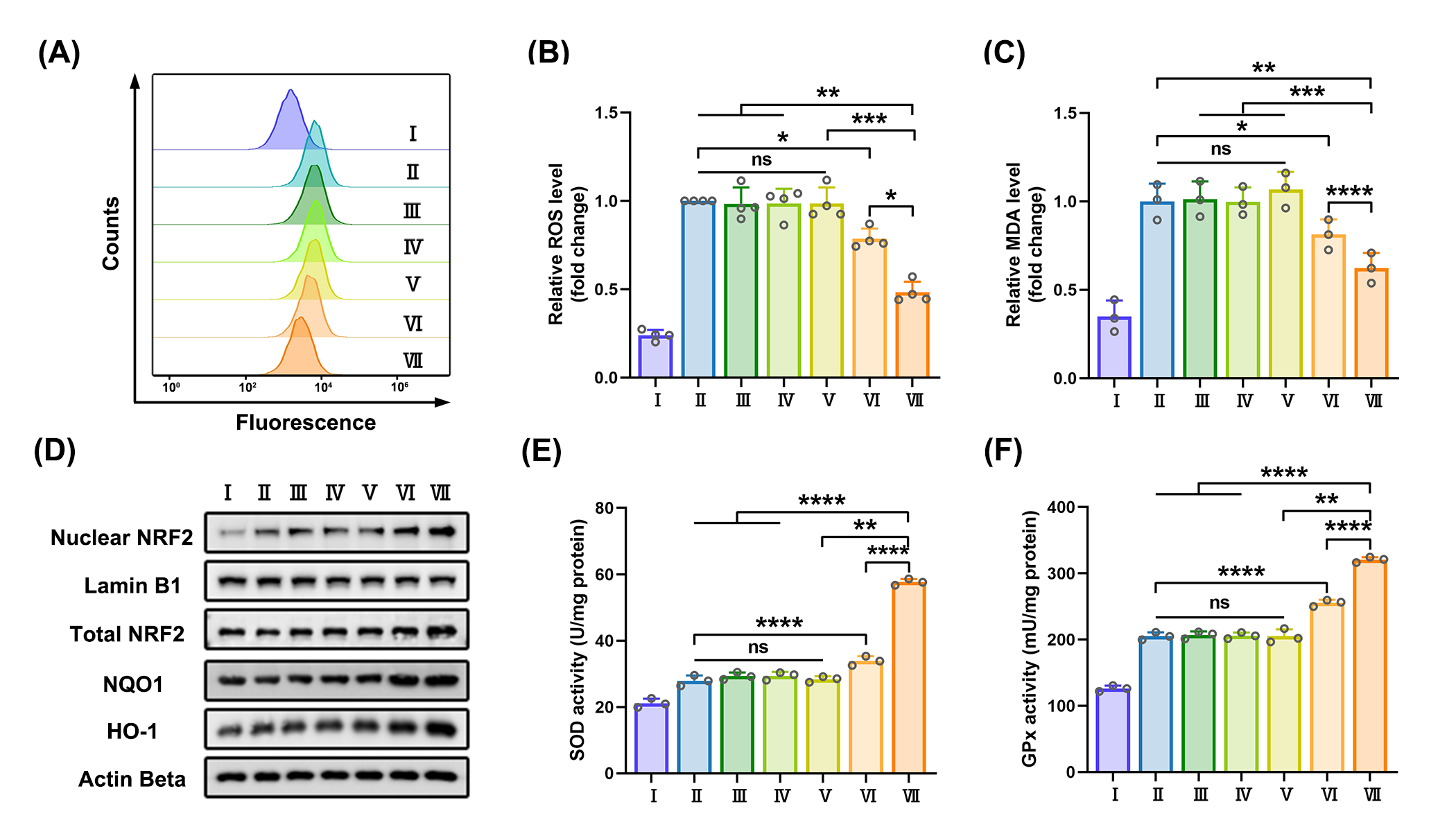


**Figure S13** MF@DeMEV/SA-MNPs alleviated oxidative stress via activating Nrf2 signaling pathway in fibroblasts. (A, B) Intracellular ROS levels in fibroblasts after different treatments measured by flow cytometry (n = 4). (C) MDA levels in fibroblasts after different treatments (n = 3). (D) Western blotting analysis of nuclear NRF2, total NRF2, NQO1 and HO-1 in fibroblasts after different treatments. Lamin B1 and Actin Beta were used as the reference genes. (E) SOD activities in fibroblasts after different treatments (n = 3). (F) GPx activities in fibroblasts after different treatments (n = 3). Data were presented as Mean ± SD; ns no significant, * p < 0.05, ** p < 0.01, *** p < 0.001, **** p < 0.0001.


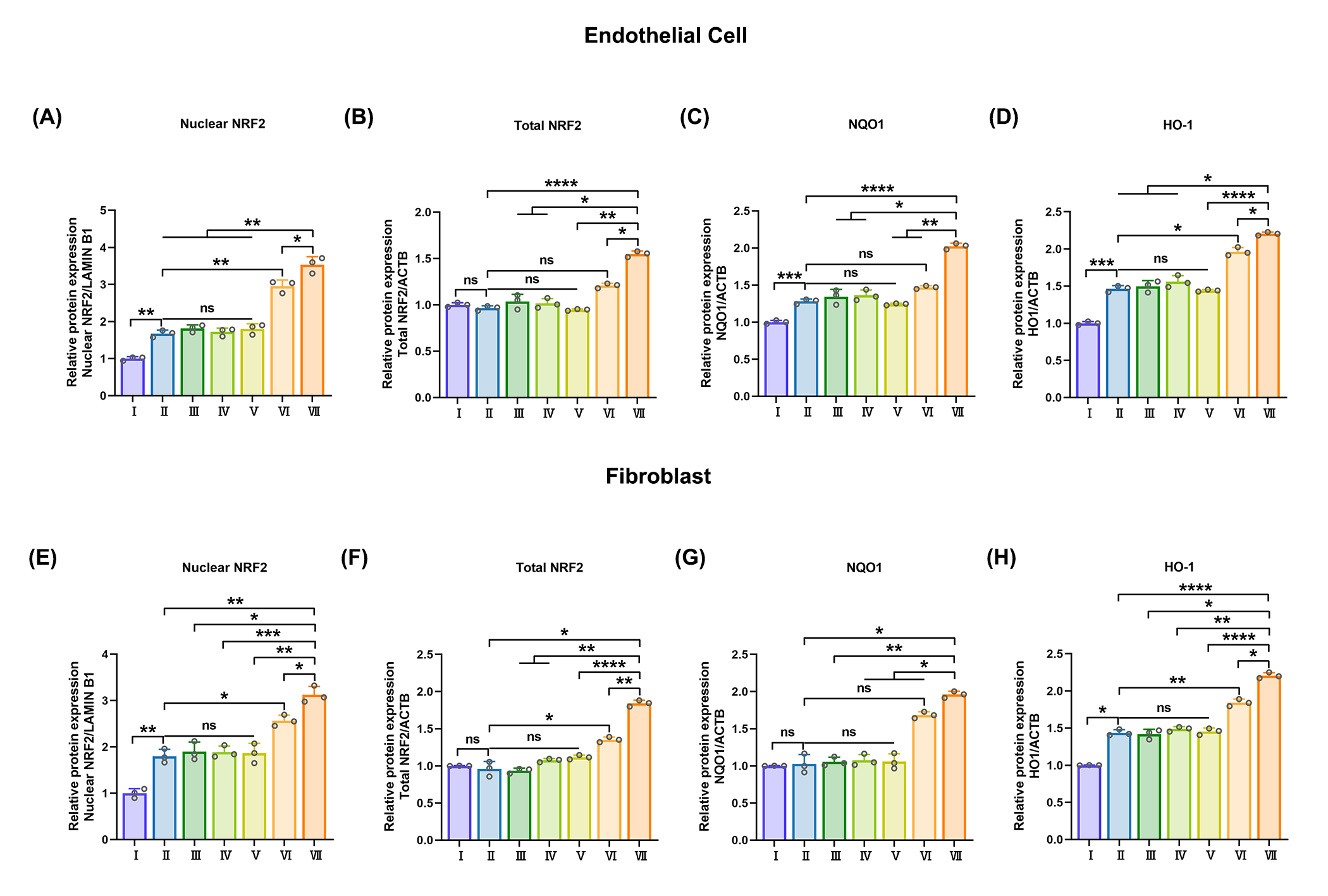


**Figure S14** Quantification analysis of western blotting analysis in Figure 5D and Figure S8D. (A-D) Quantification analysis of western blotting analysis of nuclear NRF2 (A), total NRF2 (B), NQO1 (C) and HO-1 (D) in Figure 5D (n = 3). (E-H) Quantification analysis of western blotting analysis of nuclear NRF2 (E), total NRF2 (F), NQO1 (G) and HO-1 (H) in Figure S8D (n = 3). Data were presented as Mean ± SD; ns no significant, * p < 0.05, ** p < 0.01, *** p < 0.001, **** p < 0.0001.


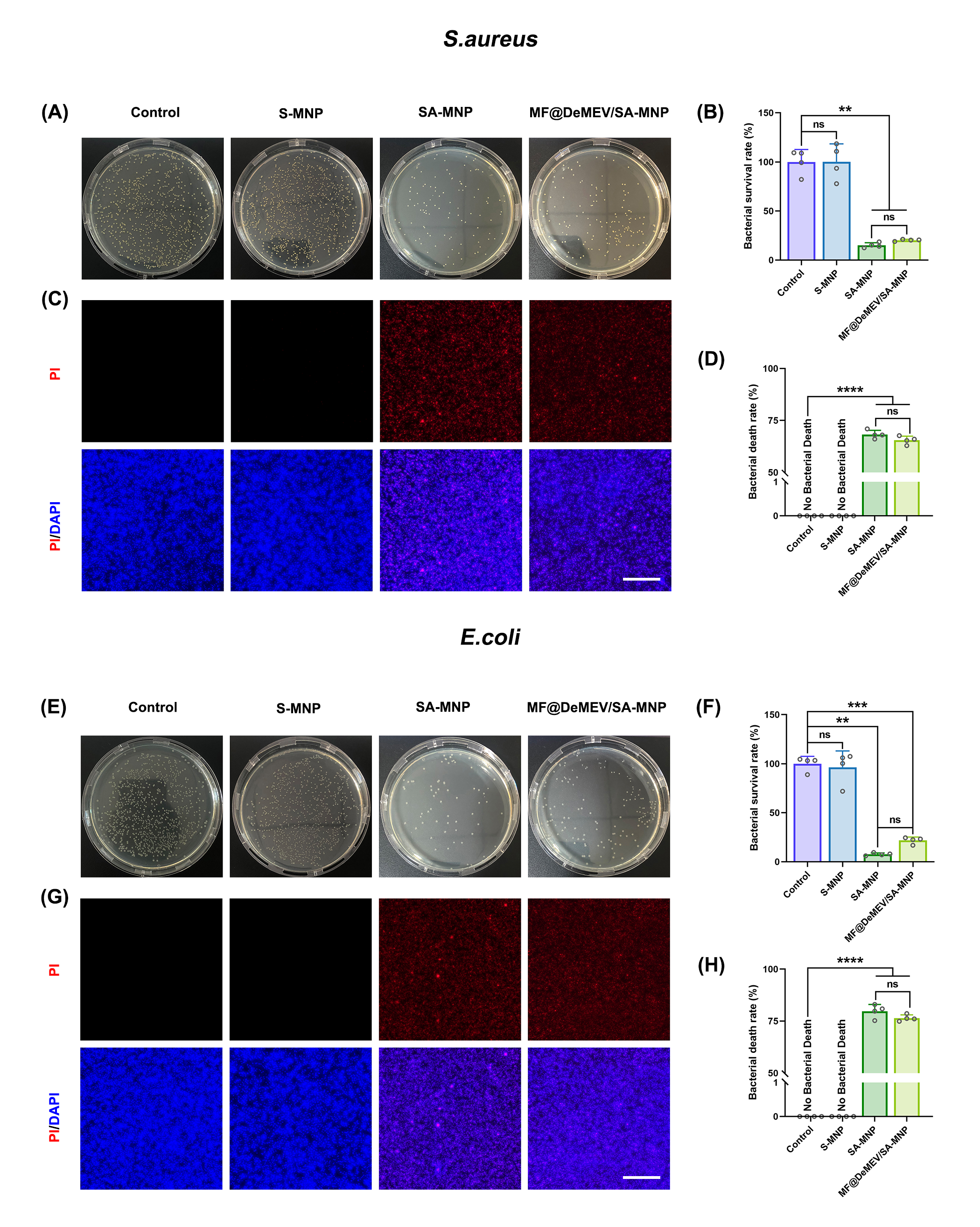


**Figure S15** Antibacterial effect of MF@DeMEV/SA-MNPs. (A, B, E, F) Colony formation of *S. aureus* (A, B) and *E. coli* (E, F) after different treatments detected by plate count method (n = 4). (C, D, G, H) Death rate of *S. aureus* (C, D) and *E. coli* (G, H) after different treatments measured by live/dead bacterial staining assay (n = 4). Scale bar: 50 μm. Data were presented as Mean ± SD; ns no significant, ** p < 0.01, *** p < 0.001, **** p < 0.0001.


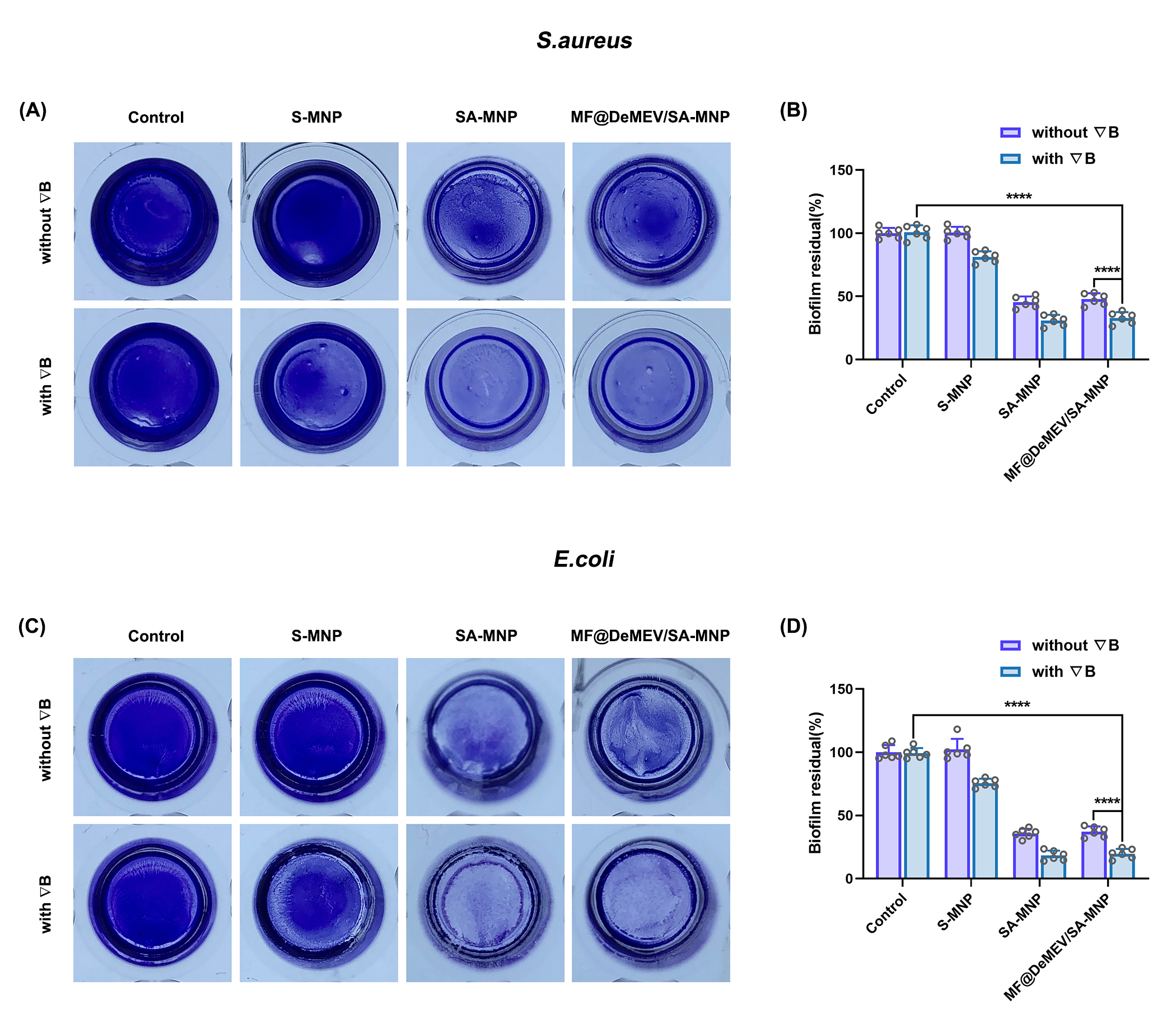


**Figure S16** Antibiofilm effect of MF@DeMEV/SA-MNPs. Biofilm residual of *S. aureus* (A, B) and *E. coli* (C, D) after different treatments measured by crystal violet-stained biofilm assay (n = 6). Data were presented as Mean ± SD; **** p < 0.0001.


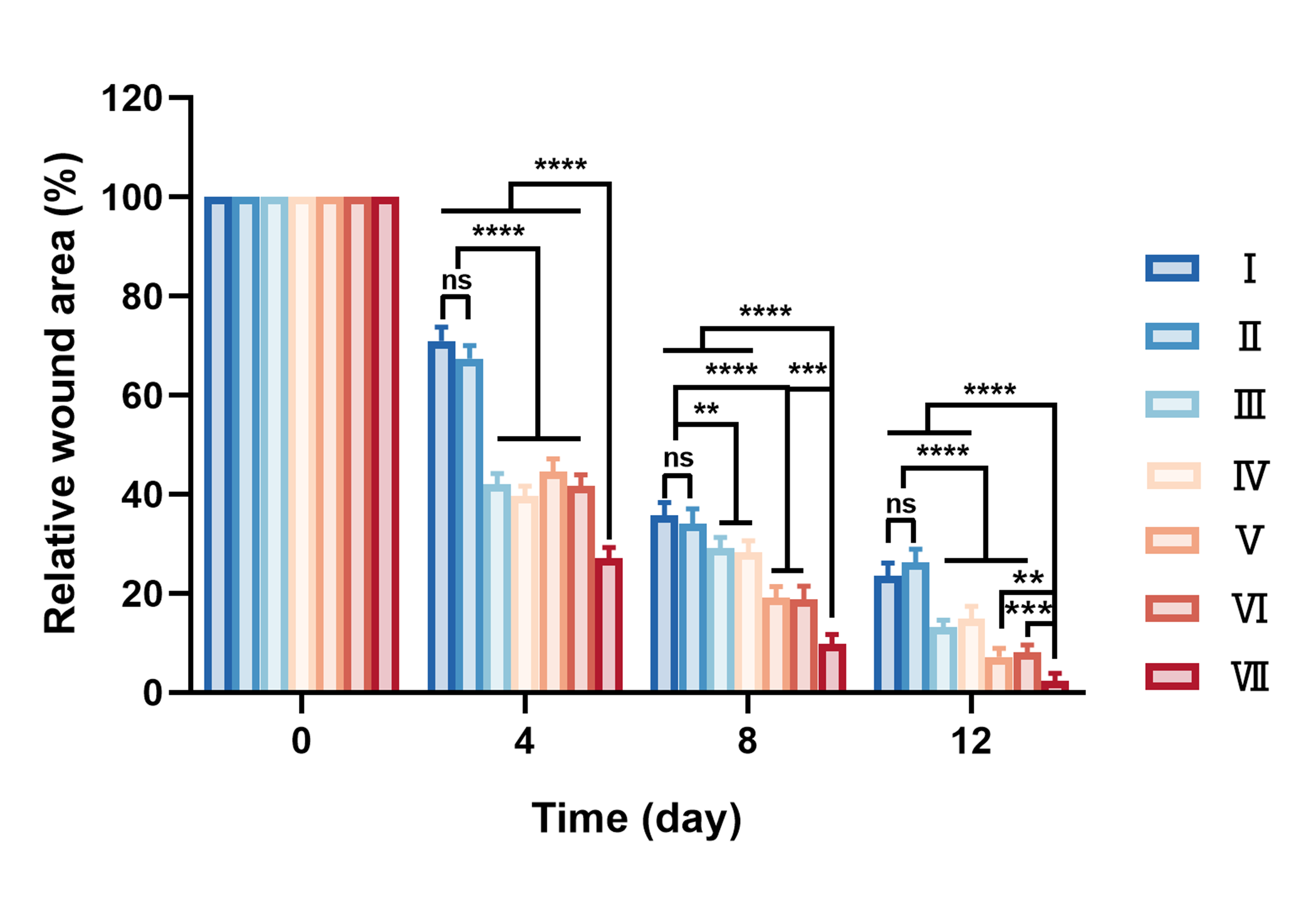


**Figure S17** Quantification analysis of the wound area in Figure 6C. The unclosed wound rate in each group at different time points (n = 6). Data were presented as Mean ± SD; ns no significant, ** p < 0.01, *** p < 0.001, **** p < 0.0001.


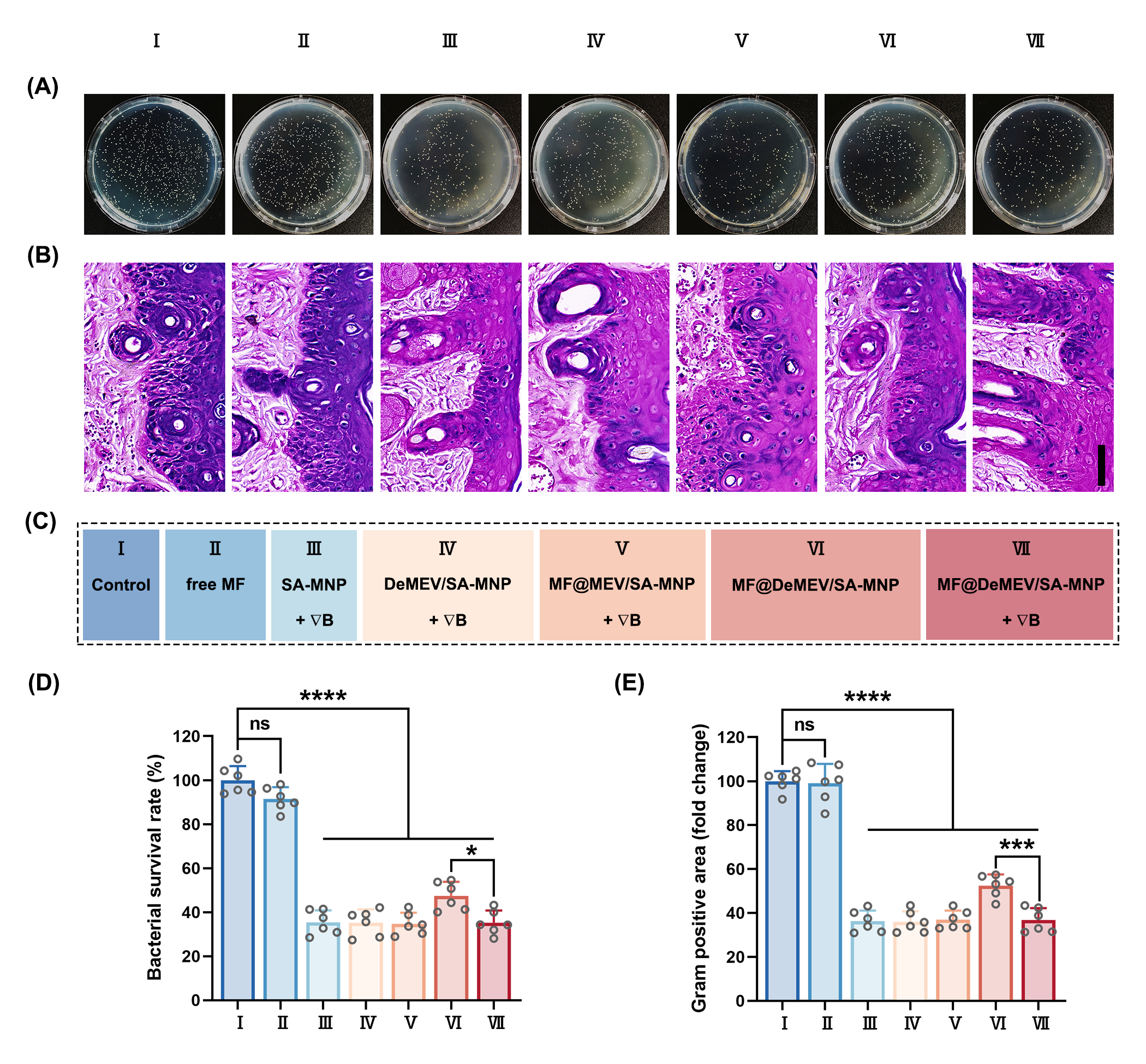


**Figure S18** Antibacterial activity of MF@DeMEV/SA-MNPs *in vivo.* (A) Representative images of *S. aureus* plate colonies obtained from infected wound tissues of mice in each group. (B) Gram staining of wound sections in each group on day 4 post-treatment. (C) The grouping information of the *in vivo* experiment. (D) Quantification analysis of bacterial survival rate in A (n = 6). (E) Quantification analysis of Gram^+^ area in B (n = 6). Scale bar: 50 μm. Data were presented as Mean ± SD; ns no significant, ** p < 0.01, *** p < 0.001, **** p < 0.0001.


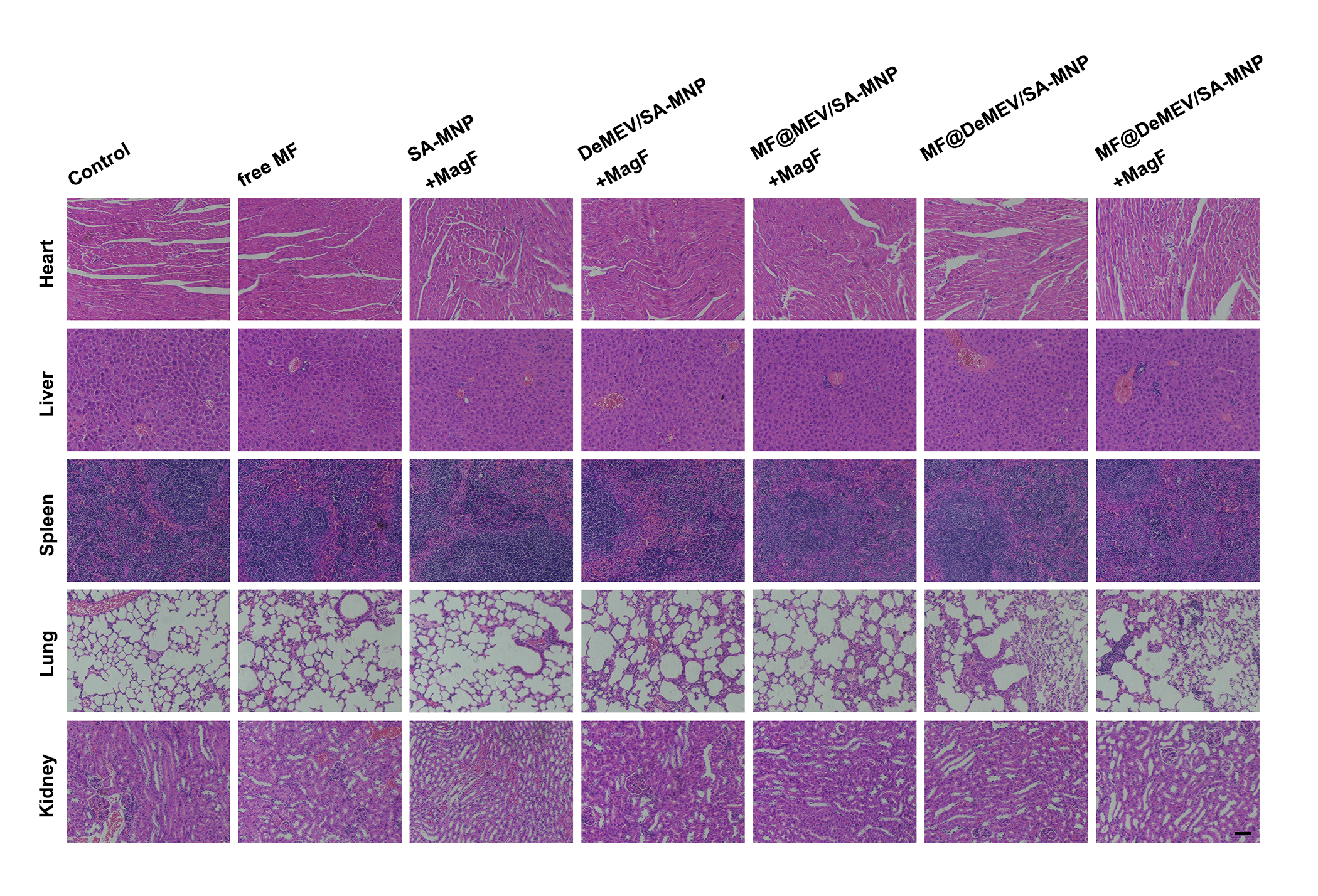


**Figure S19** *In Vivo* Biocompatibility of MF@DeMEV/SA-MNPs. H&E staining of the main organs (heart, liver, spleen, lung, and kidney) from diabetic mice with infected wounds in each group on day 12 post-treatment. Scale bar: 50 μm.

**3. Supporting movies**

**Movie S1**: Magnetic motion of biohybrid nanorobot (10 mT). 5× play speed.

**Movie S2**: Brownian motion of biohybrid nanorobot. 5× play speed.

**Movie S3**: Magnetic motion of biohybrid nanorobot swarm (10 mT). 20× play speed.

**4. References**

[1] F. Aqil, R. Munagala, J. Jeyabalan, A. K. Agrawal, A. Kyakulaga, S. A. Wilcher, R. C. Gupta, *Cancer Lett.* **2019**, *449*, 186.

[2] H. Tao, H. Xu, L. Zuo, C. Li, G. Qiao, M. Guo, L. Zheng, M. Leitgeb, X. Lin, *Int. J. Biol. Macromol.* **2020**, *161*, 470.

[3] M. B. Akolpoglu, Y. Alapan, N. O. Dogan, S. F. Baltaci, O. Yasa, G. Aybar Tural, M. Sitti, *Sci. Adv.* **2022**, *8*, eabo6163.

[4] Y. Rinkevich, G. G. Walmsley, M. S. Hu, Z. N. Maan, A. M. Newman, M. Drukker, M. Januszyk, G. W. Krampitz, G. C. Gurtner, H. P. Lorenz, I. L. Weissman, M. T. Longaker, *Science* **2015**, *348*, aaa2151.
